# Supplementary material for: Diagnosing injection-production system faults in the same well using the rough set-LVQ neural network
Source: PLoS One. 2023 Nov 27;18(11):e0291346. doi: 10.1371/journal.pone.0291346 (PMC10681231; doi:10.1371/journal.pone.0291346)
Supplement: S1 File — (ZIP) [file pone.0291346.s001.zip › A total of 770 dynamometer diagrams for 18 pumping wells/G153-443.pdf]

示 功 图 测 试 报 表

井　　号	高 153-443	测试日期	2016年 06月 03日	测试单位	试井队
矿　　名	采油七矿	仪器名称	抽油井综合测试仪	分析结果	正常

冲　　程	2.93	(m)
冲　　次	4.5	(min)
上 载 荷	63.31	(KN)
下 载 荷	27.58	(KN)
泵　　径	70	(mm)
泵　　深	986.47	(m)
杆 径 一	28	(mm)
杆 长 一	9.14	(m)

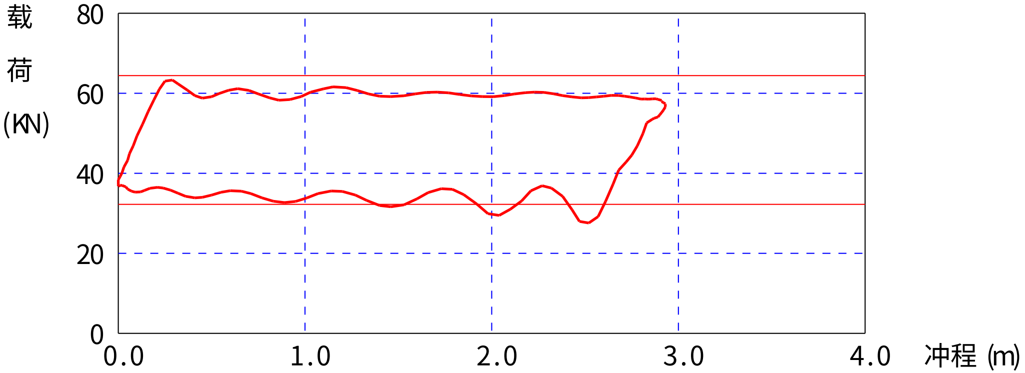

杆 径 二	25	(mm)	液 柱 重	32.18	(KN)	实际产量	55	(t)	上 电 流	27	(A)
杆 长 二	973.44	(m)	杆 柱 重	32.24	(KN)	理论排量	82.77	(t)	下 电 流	24	(A)
杆 径 三	0	(mm)	油　　压	0.6	(MPa)	含　　水	96.9	(%)	动 液 面	495.19	(m)
杆 长 三	0	(m)	套　　压	0.65	(MPa)	泵　　效	66.45	(%)	沉 没 度	491.28	(m)
测 试 人	胡　　斌		计 算 人	田 莉 梅		审 核 人	袁 莹 波		单位名称	第一采油厂	

示 功 图 测 试 报 表

井 号	高 153-443	测试日期	2016年 08月 01日	测试单位	试井队
矿 名	采油七矿	仪器名称	抽油井综合测试仪	分析结果	正常

冲 程	2.9 (m)
冲 次	4.4 (min)
上 载 荷	64.41 (KN)
下 载 荷	28.13 (KN)
泵 径	70 (mm)
泵 深	986.47 (m)
杆 径 一	28 (mm)
杆 长 一	9.14 (m)

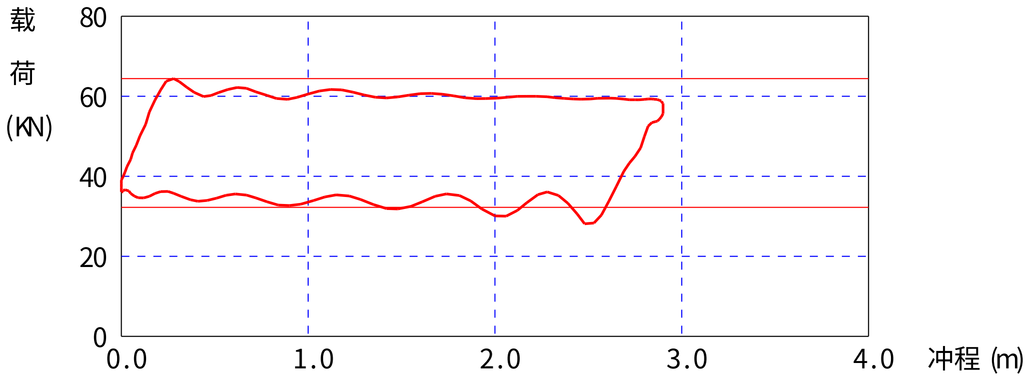

杆 径 二	25 (mm)	液 柱 重	32.18 (KN)	实际产量	64.24 (t)	上 电 流	47 (A)
杆 长 二	973.44 (m)	杆 柱 重	32.24 (KN)	理论排量	82.78 (t)	下 电 流	43 (A)
杆 径 三	0 (mm)	油 压	0.36 (MPa)	含 水	97 (%)	动 液 面	573.42 (m)
杆 长 三	0 (m)	套 压	0.44 (MPa)	泵 效	77.61 (%)	沉 没 度	413.05 (m)
测 试 人	胡 斌	计 算 人	田 莉 梅	审 核 人	袁 莹 波	单位名称	第一采油厂

示 功 图 测 试 报 表

井　　号	高 153-443		测试日期	2016年 10月 07日	测试单位	试井队	
矿　　名	采油七矿		仪器名称	抽油井综合测试仪	分析结果	正常	
冲　　程	2.91 (m)	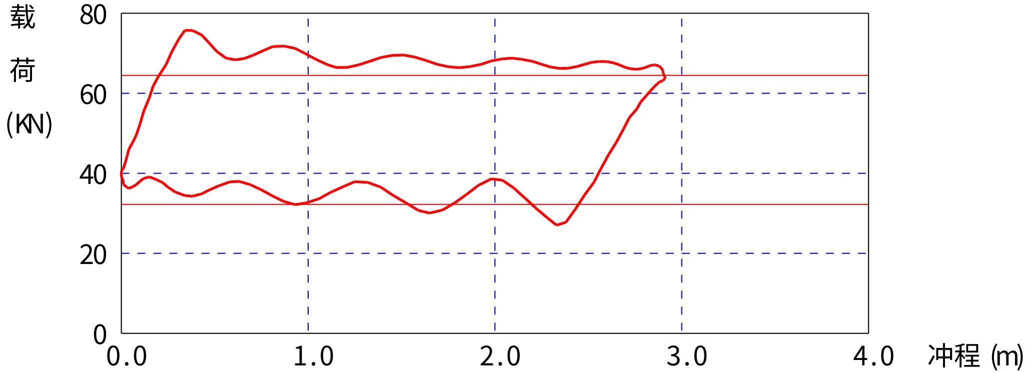					
冲　　次	5.4 (min)						
上 载 荷	75.71 (KN)						
下 载 荷	27.04 (KN)						
泵　　径	70 (mm)						
泵　　深	986.47 (m)						
杆 径 一	28 (mm)						
杆 长 一	9.14 (m)						
杆 径 二	25 (mm)	液 柱 重	32.24 (KN)	实际产量	68.07 (t)	上 电 流	60 (A)
杆 长 二	973.44 (m)	杆 柱 重	32.23 (KN)	理论排量	99.5 (t)	下 电 流	44 (A)
杆 径 三	0 (mm)	油　　压	0.51 (MPa)	含　　水	98.2 (%)	动 液 面	733.9 (m)
杆 长 三	0 (m)	套　　压	0.65 (MPa)	泵　　效	68.41 (%)	沉 没 度	252.57 (m)
测 试 人	胡　　斌	计 算 人	田 莉 梅	审 核 人	袁 莹 波	单位名称	第一采油厂

示 功 图 测 试 报 表

井 号	高 153-443	测试日期	2016年 11月 07日	测试单位	试井队
矿 名	采油七矿	仪器名称	抽油井综合测试仪	分析结果	正常

冲 程	4.95	(m)
冲 次	2.5	(min)
上 载 荷	96.88	(KN)
下 载 荷	45.1	(KN)
泵 径	40	(mm)
泵 深	702.07	(m)
杆 径 一	28	(mm)
杆 长 一	9.14	(m)

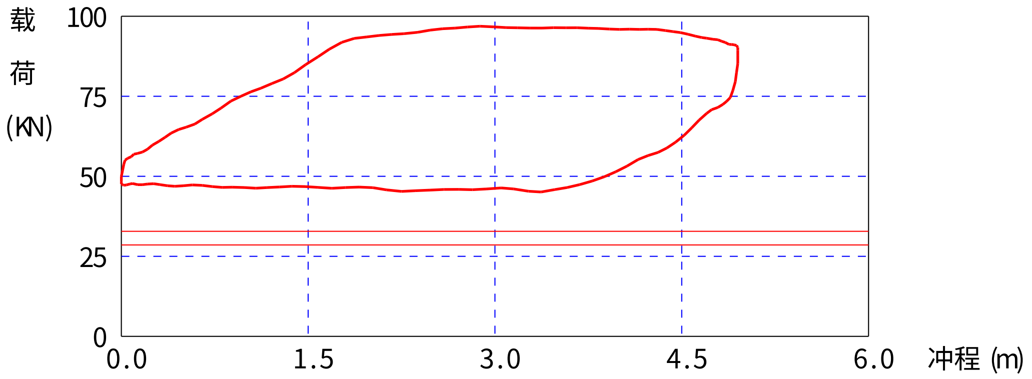

杆 径 二	28	(mm)	液 柱 重	4.26	(KN)	实际产量	14	(t)	上 电 流	99	(A)
杆 长 二	684.65	(m)	杆 柱 重	28.56	(KN)	理论排量	22.1	(t)	下 电 流	81	(A)
杆 径 三	0	(mm)	油 压	0.34	(MPa)	含 水	83.6	(%)	动 液 面	265.06	(m)
杆 长 三	0	(m)	套 压	0.43	(MPa)	泵 效	63.35	(%)	沉 没 度	437.01	(m)
测 试 人	胡 斌		计 算 人	田 莉 梅		审 核 人	袁 莹 波		单位名称	第一采油厂	

日 期 :2016-12-22 10:21 AM

示 功 图 测 试 报 表

井　　号	高 153-443	测试日期	2016年 10月 27日	测试单位	试井队
矿　　名	采油七矿	仪器名称	抽油井综合测试仪	分析结果	正常

冲　　程	4.93	(m)
冲　　次	3.5	(min)
上 载 荷	84.42	(KN)
下 载 荷	42.03	(KN)
泵　　径	70	(mm)
泵　　深	986.47	(m)
杆 径 一	28	(mm)
杆 长 一	9.14	(m)

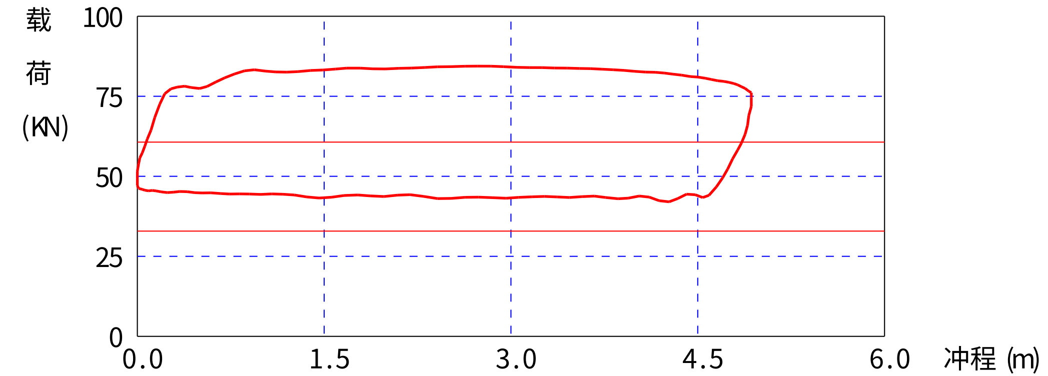

杆 径 二	25	(mm)	液 柱 重	27.8	(KN)	实际产量	0	(t)	上 电 流	0	(A)
杆 长 二	973.44	(m)	杆 柱 重	32.88	(KN)	理论排量	95.32	(t)	下 电 流	0	(A)
杆 径 三	0	(mm)	油　　压	0	(MPa)	含　　水	0	(%)	动 液 面	206.04	(m)
杆 长 三	0	(m)	套　　压	0	(MPa)	泵　　效	0	(%)	沉 没 度	780.43	(m)
测 试 人	胡　斌		计 算 人	田 莉 梅		审 核 人	袁 莹 波		单位名称	第一采油厂	

示 功 图 测 试 报 表

井 号	高 153-443	测试日期	2016年 11月 08日	测试单位	试井队
矿 名	采油七矿	仪器名称	抽油井综合测试仪	分析结果	正常

冲 程	4.95	(m)
冲 次	2.5	(min)
上 载 荷	98.57	(KN)
下 载 荷	46.17	(KN)
泵 径	40	(mm)
泵 深	702.07	(m)
杆 径 一	28	(mm)
杆 长 一	9.14	(m)

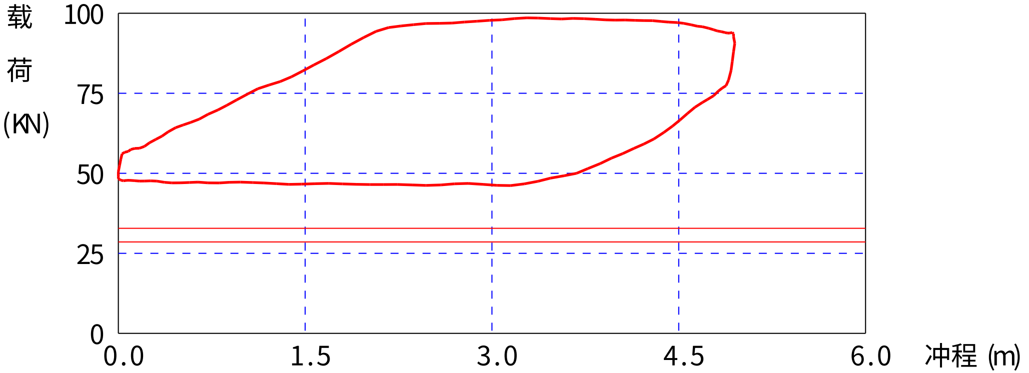

杆 径 二	28	(mm)	液 柱 重	4.26	(KN)	实际产量	15.04	(t)	上 电 流	100	(A)
杆 长 二	684.65	(m)	杆 柱 重	28.56	(KN)	理论排量	22.1	(t)	下 电 流	82	(A)
杆 径 三	0	(mm)	油 压	0.35	(MPa)	含 水	83.6	(%)	动 液 面	288.22	(m)
杆 长 三	0	(m)	套 压	0.41	(MPa)	泵 效	68.05	(%)	沉 没 度	413.85	(m)
测 试 人	胡 斌		计 算 人	田 莉 梅		审 核 人	袁 莹 波		单位名称	第一采油厂	

示 功 图 测 试 报 表

井　　号	高 153-443	测试日期	2016年 11月 18日	测试单位	试井队
矿　　名	采油七矿	仪器名称	抽油井综合测试仪	分析结果	正常

冲　　程	4.97	(m)
冲　　次	2.5	(min)
上 载 荷	99.99	(KN)
下 载 荷	42.94	(KN)
泵　　径	40	(mm)
泵　　深	702.07	(m)
杆 径 一	28	(mm)
杆 长 一	9.14	(m)

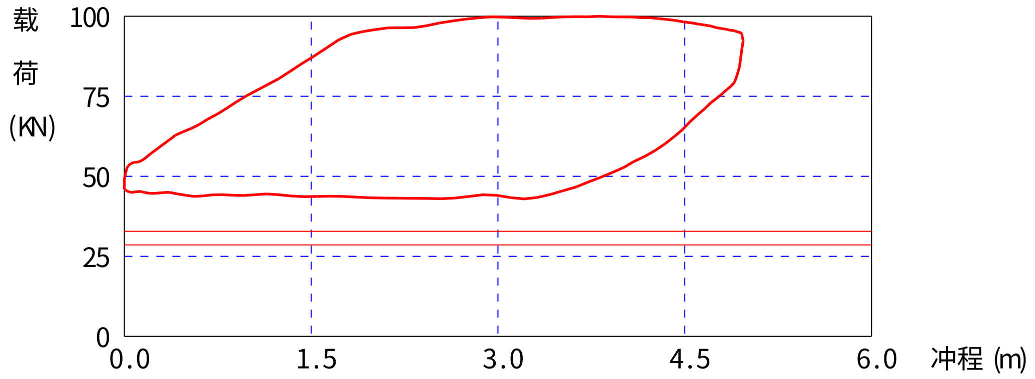

杆 径 二	28	(mm)	液 柱 重	4.25	(KN)	实际产量	6.2	(t)	上 电 流	108	(A)
杆 长 二	684.65	(m)	杆 柱 重	28.57	(KN)	理论排量	22.06	(t)	下 电 流	82	(A)
杆 径 三	0	(mm)	油　　压	0.35	(MPa)	含　　水	82.3	(%)	动 液 面	0	(m)
杆 长 三	0	(m)	套　　压	0.3	(MPa)	泵　　效	28.11	(%)	沉 没 度	702.07	(m)
测 试 人	胡　　斌		计 算 人	田 莉 梅		审 核 人	袁 莹 波		单位名称	第一采油厂	

日 期 :2016-12-22 10:21 AM

示 功 图 测 试 报 表

井 号	高 153-443	测试日期	2016年 11月 16日	测试单位	试井队
矿 名	采油七矿	仪器名称	抽油井综合测试仪	分析结果	正常

冲 程	4.97	(m)
冲 次	2.5	(min)
上 载 荷	99.24	(KN)
下 载 荷	45.26	(KN)
泵 径	40	(mm)
泵 深	702.07	(m)
杆 径 一	28	(mm)
杆 长 一	9.14	(m)

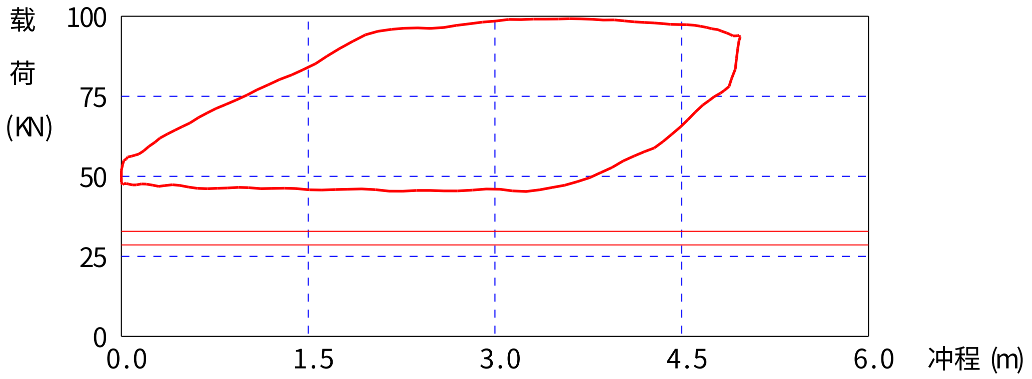

杆 径 二	28	(mm)	液 柱 重	4.26	(KN)	实际产量	7.3	(t)	上 电 流	109	(A)
杆 长 二	684.65	(m)	杆 柱 重	28.56	(KN)	理论排量	22.14	(t)	下 电 流	81	(A)
杆 径 三	0	(mm)	油 压	0.35	(MPa)	含 水	84.8	(%)	动 液 面	259.89	(m)
杆 长 三	0	(m)	套 压	0.3	(MPa)	泵 效	32.97	(%)	沉 没 度	442.18	(m)

测 试 人	胡 斌	计 算 人	田 莉 梅	审 核 人	袁 莹 波	单位名称	第一采油厂

日 期 :2016-12-22 10:21 AM

示 功 图 测 试 报 表

井　　号	高 153-443	测试日期	2016年 11月 28日	测试单位	试井队
矿　　名	采油七矿	仪器名称	抽油井综合测试仪	分析结果	正常

冲　　程	4.92	(m)
冲　　次	2.5	(min)
上 载 荷	94.87	(KN)
下 载 荷	42.51	(KN)
泵　　径	40	(mm)
泵　　深	702.07	(m)
杆 径 一	28	(mm)
杆 长 一	9.14	(m)

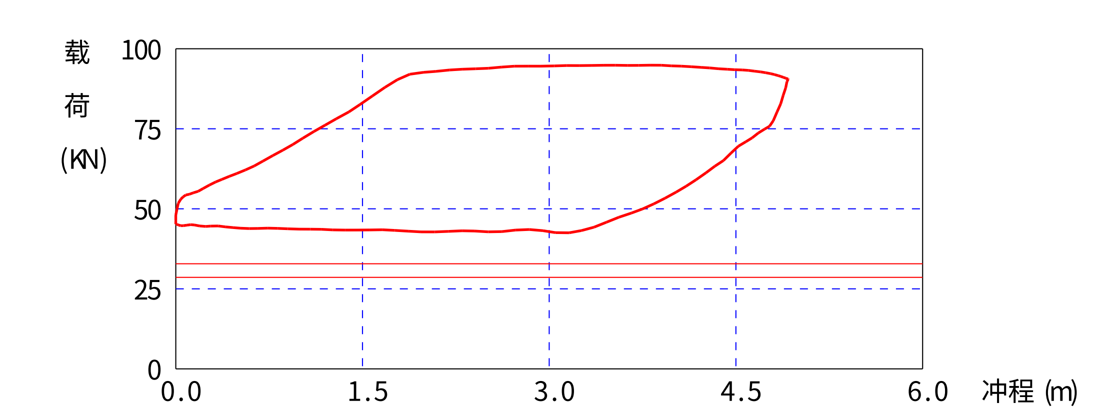

杆 径 二	28	(mm)	液 柱 重	4.25	(KN)	实际产量	5.93	(t)	上 电 流	108	(A)
杆 长 二	684.65	(m)	杆 柱 重	28.58	(KN)	理论排量	22.04	(t)	下 电 流	84	(A)
杆 径 三	0	(mm)	油　　压	0.38	(MPa)	含　　水	81.6	(%)	动 液 面	-1	(m)
杆 长 三	0	(m)	套　　压	0.22	(MPa)	泵　　效	26.91	(%)	沉 没 度	0	(m)
测 试 人	胡　　斌		计 算 人	田 莉 梅		审 核 人	袁 莹 波		单位名称	第一采油厂	

日 期 :2016-12-22 10:21 AM

示 功 图 测 试 报 表

井号	高 153-443	测试日期	2016年 11月 24日	测试单位	试井队
矿名	采油七矿	仪器名称	抽油井综合测试仪	分析结果	正常

冲程	4.93	(m)
冲次	2.5	(min)
上载荷	97.03	(KN)
下载荷	42.63	(KN)
泵径	40	(mm)
泵深	702.07	(m)
杆径一	28	(mm)
杆长一	9.14	(m)

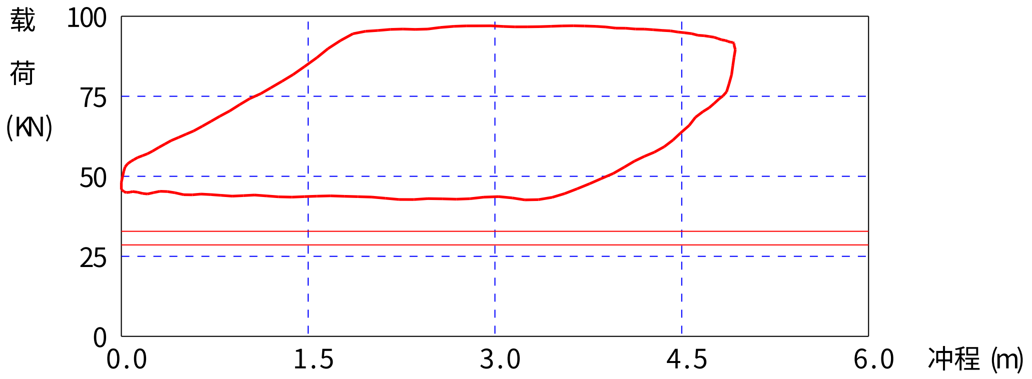

杆径二	28	(mm)	液柱重	4.25	(KN)	实际产量	6.87	(t)	上电流	108	(A)
杆长二	684.65	(m)	杆柱重	28.57	(KN)	理论排量	22.06	(t)	下电流	85	(A)
杆径三	0	(mm)	油压	0.4	(MPa)	含水	82.3	(%)	动液面	-1	(m)
杆长三	0	(m)	套压	0.34	(MPa)	泵效	31.14	(%)	沉没度	0	(m)
测试人	胡 斌		计算人	田莉梅		审核人	袁莹波		单位名称	第一采油厂	

日 期 :2016-12-22 10:21 AM

示 功 图 测 试 报 表

井　　号	高 153-443	测试日期	2016年 11月 30日	测试单位	试井队
矿　　名	采油七矿	仪器名称	抽油井综合测试仪	分析结果	正常

冲　　程	4.93	(m)
冲　　次	1.9	(min)
上 载 荷	87.42	(KN)
下 载 荷	40.9	(KN)
泵　　径	40	(mm)
泵　　深	702.07	(m)
杆 径 一	28	(mm)
杆 长 一	9.14	(m)

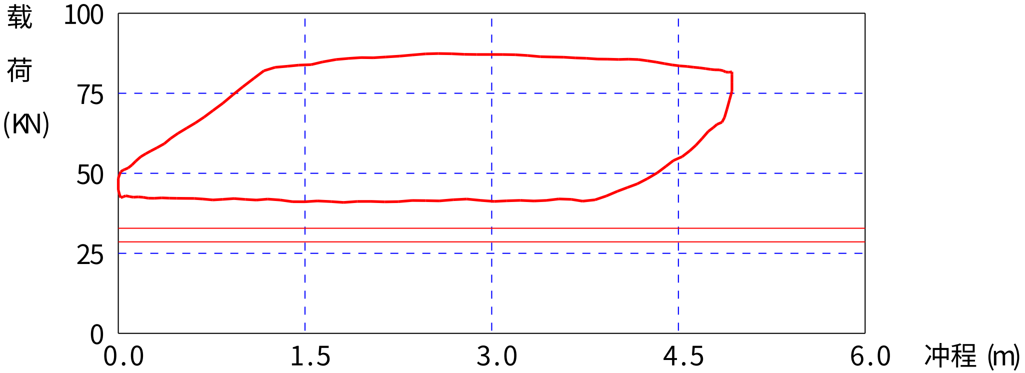

杆 径 二	28	(mm)	液 柱 重	4.25	(KN)	实际产量	0	(t)	上 电 流	108	(A)
杆 长 二	684.65	(m)	杆 柱 重	28.58	(KN)	理论排量	22.04	(t)	下 电 流	84	(A)
杆 径 三	0	(mm)	油　　压	0.37	(MPa)	含　　水	81.6	(%)	动 液 面	85.43	(m)
杆 长 三	0	(m)	套　　压	0.17	(MPa)	泵　　效	0	(%)	沉 没 度	616.64	(m)
测 试 人	胡　斌		计 算 人	田 莉 梅		审 核 人	袁 莹 波		单位名称	第一采油厂	

示 功 图 测 试 报 表

井　　号	高 153-443	测试日期	2016年 12月 19日	测试单位	试井队
矿　　名	采油七矿	仪器名称	抽油井综合测试仪	分析结果	正常

冲　　程	4.98	(m)
冲　　次	1.9	(min)
上 载 荷	87.19	(KN)
下 载 荷	39.62	(KN)
泵　　径	40	(mm)
泵　　深	702.07	(m)
杆 径 一	28	(mm)
杆 长 一	9.14	(m)

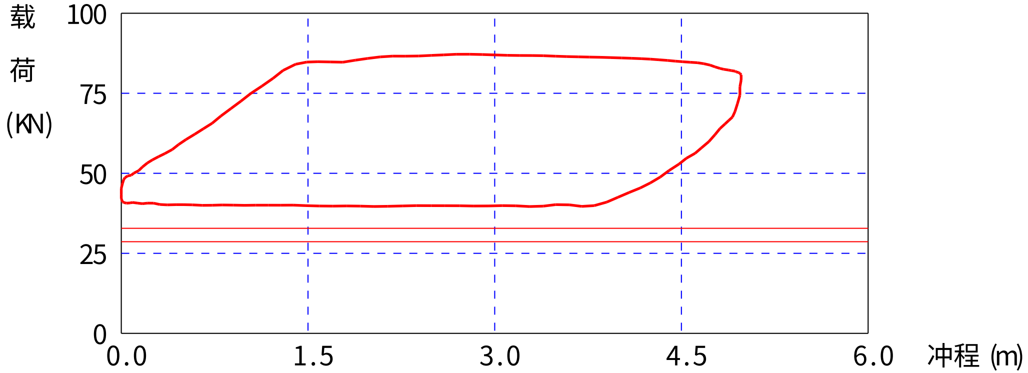

杆 径 二	28	(mm)	液 柱 重	4.19	(KN)	实际产量	7.23	(t)	上 电 流	95	(A)
杆 长 二	684.65	(m)	杆 柱 重	28.63	(KN)	理论排量	16.52	(t)	下 电 流	83	(A)
杆 径 三	0	(mm)	油　　压	0.34	(MPa)	含　　水	72.2	(%)	动 液 面	91.02	(m)
杆 长 三	0	(m)	套　　压	0.27	(MPa)	泵　　效	43.76	(%)	沉 没 度	611.05	(m)
测 试 人	胡　　斌		计 算 人	田 莉 梅		审 核 人	袁 莹 波		单位名称	第一采油厂	

日 期 :2016-12-22 10:21 AM

示 功 图 测 试 报 表

井 号	高 153-443	测试日期	2016年 02月 16日	测试单位	五零三队
矿 名	采油七矿	仪器名称	电脑测井仪	分析结果	正常

冲 程	2.92	(m)
冲 次	4.5	(min)
上 载 荷	67.48	(KN)
下 载 荷	29.43	(KN)
泵 径	70	(mm)
泵 深	986.47	(m)
杆 径 一	28	(mm)
杆 长 一	9.14	(m)

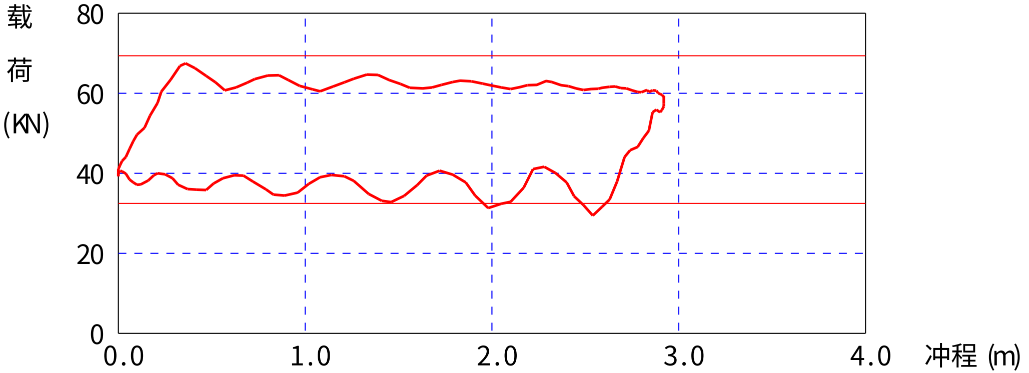

杆 径 二	25	(mm)	液 柱 重	36.91	(KN)	实际产量	21.35	(t)	上 电 流	29	(A)
杆 长 二	973.44	(m)	杆 柱 重	32.47	(KN)	理论排量	72.6	(t)	下 电 流	26	(A)
杆 径 三		(mm)	油 压	0.5	(MPa)	含 水	97.1	(%)	动 液 面	598.17	(m)
杆 长 三	0	(m)	套 压	0.56	(MPa)	泵 效	29.4	(%)	沉 没 度	388.3	(m)
测 试 人	张 恕 涛		计 算 人	田 莉 梅		审 核 人	袁 莹 波		单位名称	第一采油厂	

示 功 图 测 试 报 表

井号	高 153-443	测试日期	2016年 04月 05日	测试单位	试井队
矿名	采油七矿	仪器名称	抽油井综合测试仪	分析结果	正常

冲程	2.97	(m)
冲次	4.5	(min)
上载荷	63.41	(KN)
下载荷	25.15	(KN)
泵径	70	(mm)
泵深	986.47	(m)
杆径一	28	(mm)
杆长一	9.14	(m)

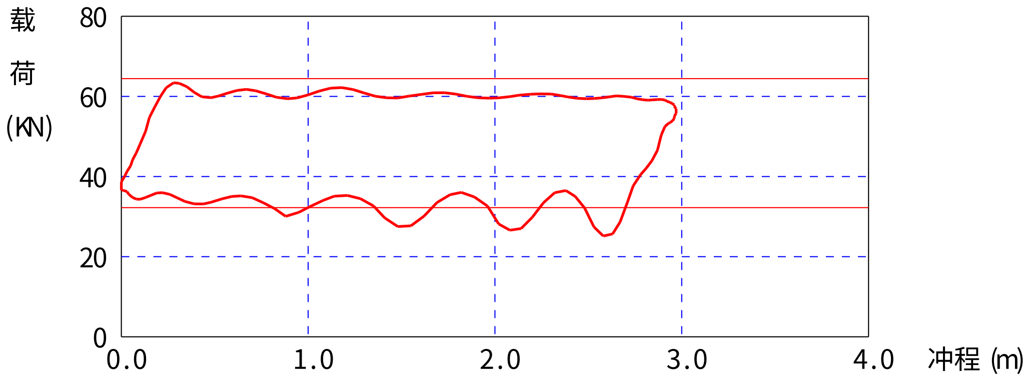

杆径二	25	(mm)	液柱重	32.2	(KN)	实际产量	62.04	(t)	上电流	30	(A)
杆长二	973.44	(m)	杆柱重	32.24	(KN)	理论排量	82.82	(t)	下电流	27	(A)
杆径三	0	(mm)	油压	0.56	(MPa)	含水	97.4	(%)	动液面	499.15	(m)
杆长三	0	(m)	套压	0.6	(MPa)	泵效	74.91	(%)	沉没度	487.32	(m)
测试人	胡 斌		计算人	田莉梅		审核人	袁莹波		单位名称	第一采油厂	

日 期 :2016-12-22 10:21 AM

示 功 图 测 试 报 表

井 号	高 153-443	测试日期	2016年 05月 13日	测试单位	试井队
矿 名	采油七矿	仪器名称	抽油井综合测试仪	分析结果	正常

冲 程	2.91	(m)
冲 次	4.5	(min)
上 载 荷	62.58	(KN)
下 载 荷	26.5	(KN)
泵 径	70	(mm)
泵 深	986.47	(m)
杆 径 一	28	(mm)
杆 长 一	9.14	(m)

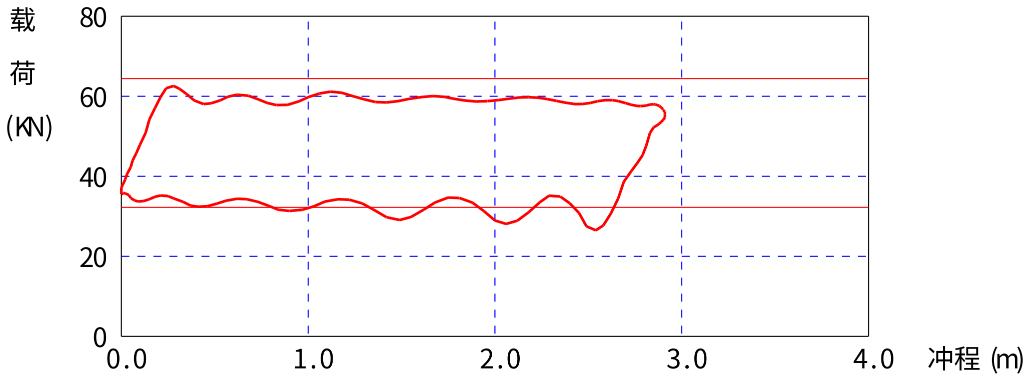

杆 径 二	25	(mm)	液 柱 重	32.18	(KN)	实际产量	69.38	(t)	上 电 流	28	(A)
杆 长 二	973.44	(m)	杆 柱 重	32.24	(KN)	理论排量	82.78	(t)	下 电 流	25	(A)
杆 径 三	0	(mm)	油 压	0.48	(MPa)	含 水	97	(%)	动 液 面	532.87	(m)
杆 长 三	0	(m)	套 压	0.5	(MPa)	泵 效	83.82	(%)	沉 没 度	453.6	(m)
测 试 人	胡 斌		计 算 人	田 莉 梅		审 核 人	袁 莹 波		单位名称	第一采油厂	

日 期 :2016-12-22 10:21 AM

示　功　图　测　试　报　表

井　　号	高 153-443	测试日期	2016年 08月 09日	测试单位	试井队
矿　　名	采油七矿	仪器名称	抽油井综合测试仪	分析结果	正常

冲　　程	2.94	(m)
冲　　次	5.3	(min)
上 载 荷	71.3	(KN)
下 载 荷	25.01	(KN)
泵　　径	70	(mm)
泵　　深	986.47	(m)
杆 径 一	28	(mm)
杆 长 一	9.14	(m)

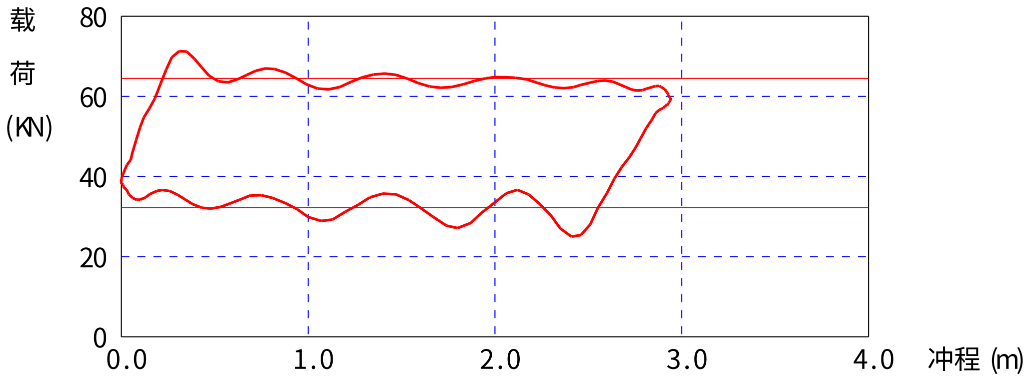

杆 径 二	25	(mm)	液 柱 重	32.25	(KN)	实际产量	63.87	(t)	上 电 流	48	(A)
杆 长 二	973.44	(m)	杆 柱 重	32.23	(KN)	理论排量	99.54	(t)	下 电 流	43	(A)
杆 径 三	0	(mm)	油　　压	0.37	(MPa)	含　　水	98.5	(%)	动 液 面	623.14	(m)
杆 长 三	0	(m)	套　　压	0.47	(MPa)	泵　　效	64.16	(%)	沉 没 度	363.33	(m)
测 试 人	胡　斌		计 算 人	田 莉 梅		审 核 人	袁 莹 波		单位名称	第一采油厂	

日 期 :2016-12-22 10:21 AM

示 功 图 测 试 报 表

井　　号	高 153-443	测试日期	2016年 11月 02日	测试单位	试井队
矿　　名	采油七矿	仪器名称	抽油井综合测试仪	分析结果	正常

冲　　程	4.99 (m)
冲　　次	2.5 (min)
上 载 荷	93.11 (KN)
下 载 荷	47.69 (KN)
泵　　径	70 (mm)
泵　　深	986.47 (m)
杆 径 一	28 (mm)
杆 长 一	9.14 (m)

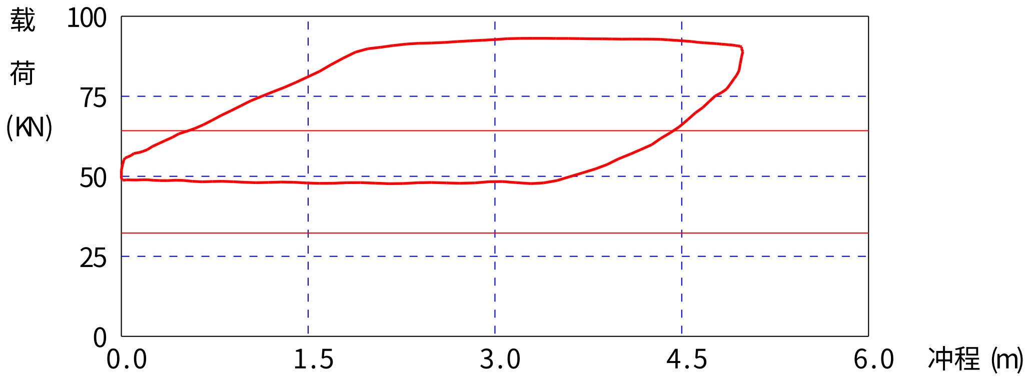

杆 径 二	25 (mm)	液 柱 重	31.98 (KN)	实际产量	12.46 (t)	上 电 流	96 (A)
杆 长 二	973.44 (m)	杆 柱 重	32.27 (KN)	理论排量	68.54 (t)	下 电 流	84 (A)
杆 径 三	0 (mm)	油　　压	0.33 (MPa)	含　　水	92.4 (%)	动 液 面	322.9 (m)
杆 长 三	0 (m)	套　　压	0.31 (MPa)	泵　　效	18.18 (%)	沉 没 度	663.57 (m)
测 试 人	胡　斌	计 算 人	田 莉 梅	审 核 人	袁 莹 波	单位名称	第一采油厂

日 期 :2016-12-22 10:21 AM

示 功 图 测 试 报 表

井　　号	高 153-443	测试日期	2016年 11月 14日	测试单位	试井队
矿　　名	采油七矿	仪器名称	抽油井综合测试仪	分析结果	正常

冲　　程	4.9	(m)
冲　　次	2.6	(min)
上 载 荷	93.17	(KN)
下 载 荷	42.63	(KN)
泵　　径	40	(mm)
泵　　深	702.07	(m)
杆 径 一	28	(mm)
杆 长 一	9.14	(m)

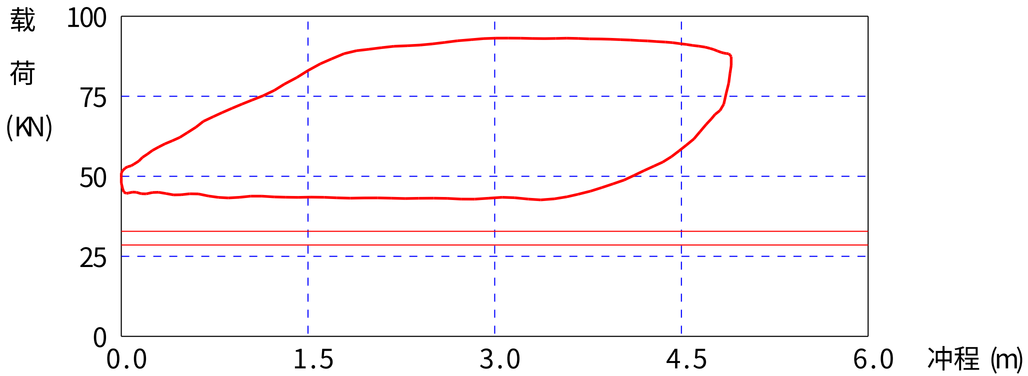

杆 径 二	28	(mm)	液 柱 重	4.28	(KN)	实际产量	13.58	(t)	上 电 流	103	(A)
杆 长 二	684.65	(m)	杆 柱 重	28.54	(KN)	理论排量	22.23	(t)	下 电 流	79	(A)
杆 径 三	0	(mm)	油　　压	0.35	(MPa)	含　　水	87.8	(%)	动 液 面	273.6	(m)
杆 长 三	0	(m)	套　　压	0.3	(MPa)	泵　　效	61.08	(%)	沉 没 度	428.48	(m)
测 试 人	胡　斌		计 算 人	田 莉 梅		审 核 人	袁 莹 波		单位名称	第一采油厂	

示 功 图 测 试 报 表

井号	高153-443	测试日期	2016年11月15日	测试单位	试井队
矿名	采油七矿	仪器名称	抽油井综合测试仪	分析结果	正常

冲程	4.95	(m)
冲次	2.5	(min)
上载荷	92.29	(KN)
下载荷	44.64	(KN)
泵径	40	(mm)
泵深	702.07	(m)
杆径一	28	(mm)
杆长一	9.14	(m)

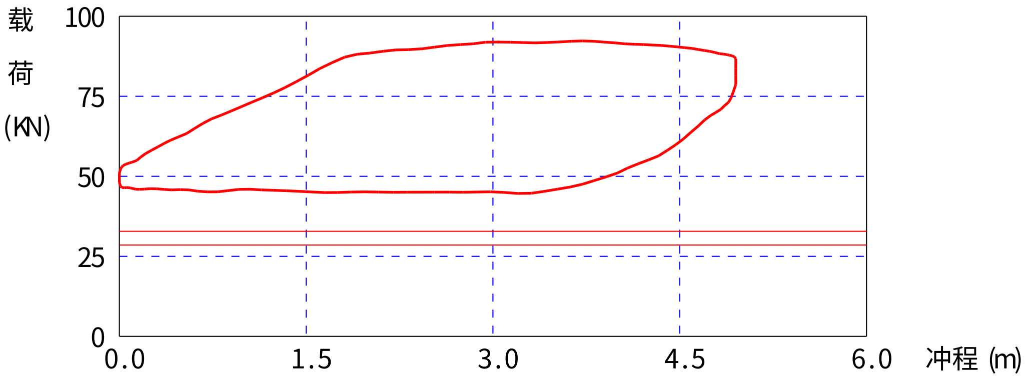

杆径二	28	(mm)	液柱重	4.29	(KN)	实际产量	9.68	(t)	上电流	108	(A)
杆长二	684.65	(m)	杆柱重	28.53	(KN)	理论排量	22.26	(t)	下电流	80	(A)
杆径三	0	(mm)	油压	0.34	(MPa)	含水	88.8	(%)	动液面	284.86	(m)
杆长三	0	(m)	套压	0.31	(MPa)	泵效	43.48	(%)	沉没度	417.21	(m)
测试人	胡 斌		计算人	田莉梅		审核人	袁莹波		单位名称	第一采油厂	

日 期 :2016-12-22 10:21 AM

示 功 图 测 试 报 表

井 号	高 153-443	测试日期	2016年 11月 29日	测试单位	试井队
矿 名	采油七矿	仪器名称	抽油井综合测试仪	分析结果	正常

冲 程	4.99	(m)
冲 次	2.5	(min)
上 载 荷	70.07	(KN)
下 载 荷	36.17	(KN)
泵 径	40	(mm)
泵 深	702.07	(m)
杆 径 一	28	(mm)
杆 长 一	9.14	(m)

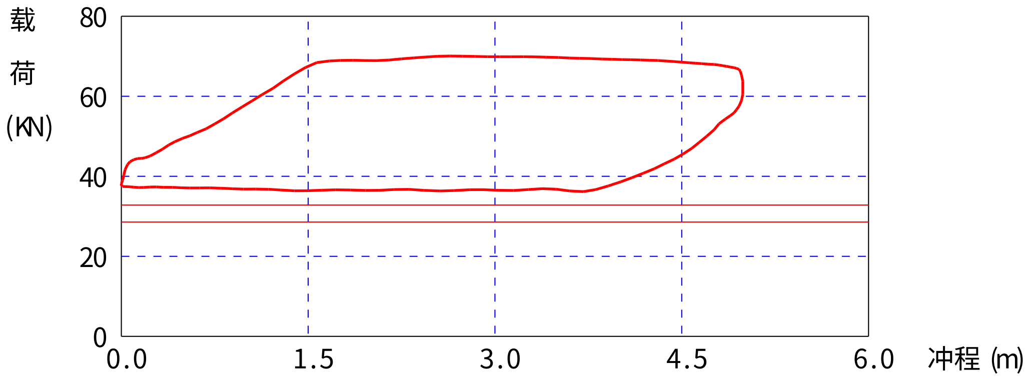

杆 径 二	28	(mm)	液 柱 重	4.25	(KN)	实际产量	0	(t)	上 电 流	109	(A)
杆 长 二	684.65	(m)	杆 柱 重	28.58	(KN)	理论排量	22.04	(t)	下 电 流	85	(A)
杆 径 三	0	(mm)	油 压	0.37	(MPa)	含 水	81.6	(%)	动 液 面	176.98	(m)
杆 长 三	0	(m)	套 压	0.21	(MPa)	泵 效	0	(%)	沉 没 度	525.09	(m)
测 试 人	胡 斌		计 算 人	田 莉 梅		审 核 人	袁 莹 波		单位名称	第一采油厂	

日 期 :2016-12-22 10:21 AM

示 功 图 测 试 报 表

井 号	高 153-443	测试日期	2016年 11月 17日	测试单位	试井队
矿 名	采油七矿	仪器名称	抽油井综合测试仪	分析结果	正常

冲 程	4.96	(m)
冲 次	2.5	(min)
上 载 荷	95.43	(KN)
下 载 荷	41.85	(KN)
泵 径	40	(mm)
泵 深	702.07	(m)
杆 径 一	28	(mm)
杆 长 一	9.14	(m)

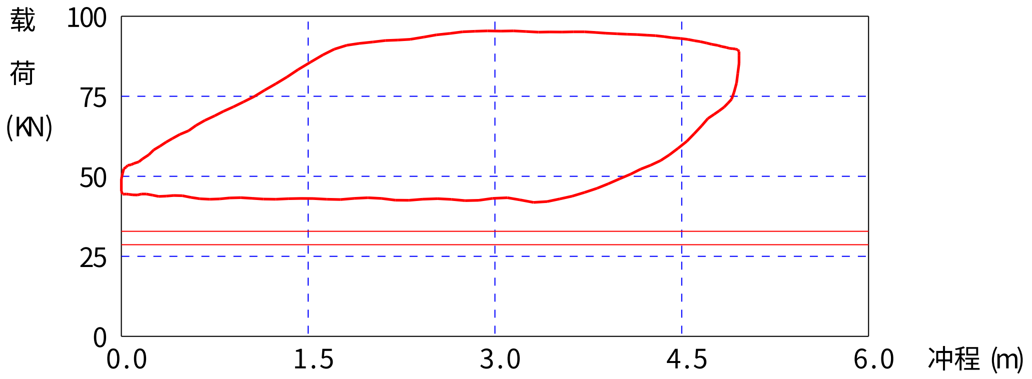

杆 径 二	28	(mm)	液 柱 重	4.19	(KN)	实际产量	6.6	(t)	上 电 流	110	(A)
杆 长 二	684.65	(m)	杆 柱 重	28.63	(KN)	理论排量	21.76	(t)	下 电 流	81	(A)
杆 径 三	0	(mm)	油 压	0.34	(MPa)	含 水	72.8	(%)	动 液 面	264.03	(m)
杆 长 三	0	(m)	套 压	0.28	(MPa)	泵 效	30.33	(%)	沉 没 度	438.04	(m)
测 试 人	胡 斌		计 算 人	田 莉 梅		审 核 人	袁 莹 波		单位名称	第一采油厂	

日 期:2016-12-22 10:21 AM

示 功 图 测 试 报 表

井　　号	高 153-443	测试日期	2016年 12月 09日	测试单位	试井队
矿　　名	采油七矿	仪器名称	抽油井综合测试仪	分析结果	正常

冲　　程	4.98	(m)
冲　　次	1.9	(min)
上 载 荷	88.04	(KN)
下 载 荷	38.46	(KN)
泵　　径	40	(mm)
泵　　深	702.07	(m)
杆 径 一	28	(mm)
杆 长 一	9.14	(m)

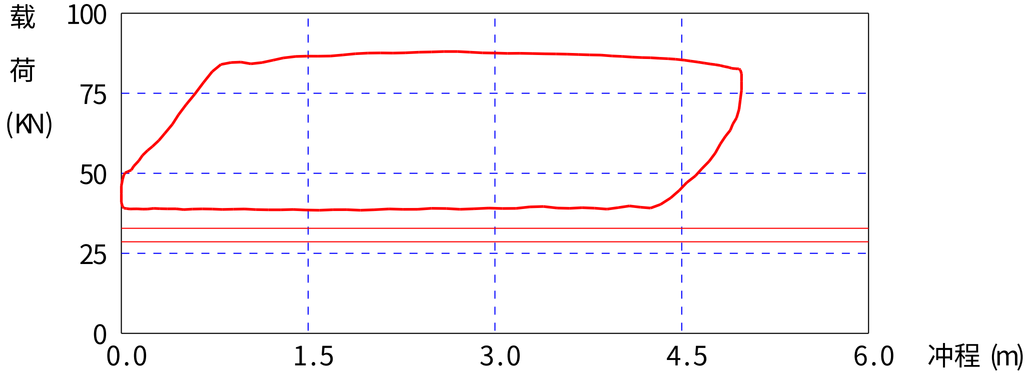

杆 径 二	28	(mm)	液 柱 重	4.21	(KN)	实际产量	5.65	(t)	上 电 流	96	(A)
杆 长 二	684.65	(m)	杆 柱 重	28.61	(KN)	理论排量	16.61	(t)	下 电 流	82	(A)
杆 径 三	0	(mm)	油　　压	0.4	(MPa)	含　　水	75.9	(%)	动 液 面	86.64	(m)
杆 长 三	0	(m)	套　　压	0.3	(MPa)	泵　　效	34.01	(%)	沉 没 度	615.43	(m)
测 试 人	胡　斌		计 算 人	田 莉 梅		审 核 人	袁 莹 波		单位名称	第一采油厂	

示 功 图 测 试 报 表

井　　号	高 153-443	测试日期	2016年 12月 12日	测试单位	试井队
矿　　名	采油七矿	仪器名称	抽油井综合测试仪	分析结果	正常

冲　　程	4.98	(m)
冲　　次	1.9	(min)
上 载 荷	86.43	(KN)
下 载 荷	38.9	(KN)
泵　　径	40	(mm)
泵　　深	702.07	(m)
杆 径 一	28	(mm)
杆 长 一	9.14	(m)

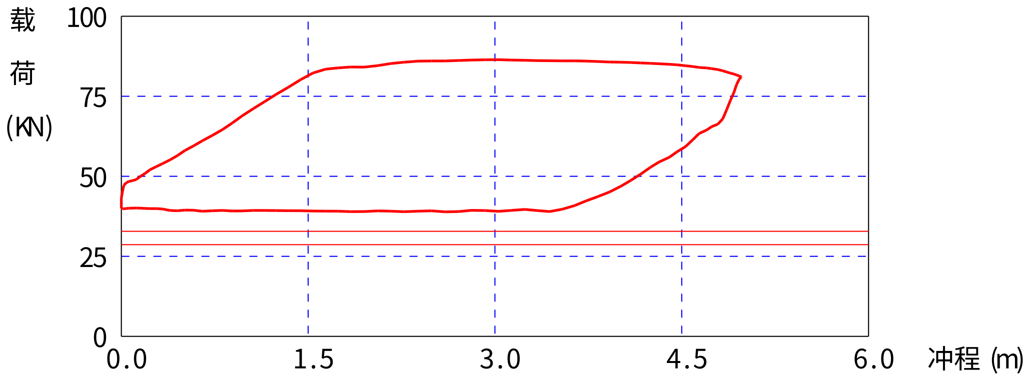

杆 径 二	28	(mm)	液 柱 重	4.17	(KN)	实际产量	7.5	(t)	上 电 流	95	(A)
杆 长 二	684.65	(m)	杆 柱 重	28.65	(KN)	理论排量	16.44	(t)	下 电 流	84	(A)
杆 径 三	0	(mm)	油　　压	0.36	(MPa)	含　　水	68.9	(%)	动 液 面	0	(m)
杆 长 三	0	(m)	套　　压	0.35	(MPa)	泵　　效	45.61	(%)	沉 没 度	702.07	(m)
测 试 人	胡　　斌		计 算 人	田 莉 梅		审 核 人	袁 莹 波		单位名称	第一采油厂	

日 期 :2016-12-22 10:21 AM

示 功 图 测 试 报 表

井 号	高 153-443	测试日期	2016年 12月 08日	测试单位	试井队
矿 名	采油七矿	仪器名称	抽油井综合测试仪	分析结果	正常

冲 程	4.98	(m)
冲 次	1.9	(min)
上 载 荷	90.08	(KN)
下 载 荷	40.72	(KN)
泵 径	40	(mm)
泵 深	702.07	(m)
杆 径 一	28	(mm)
杆 长 一	9.14	(m)

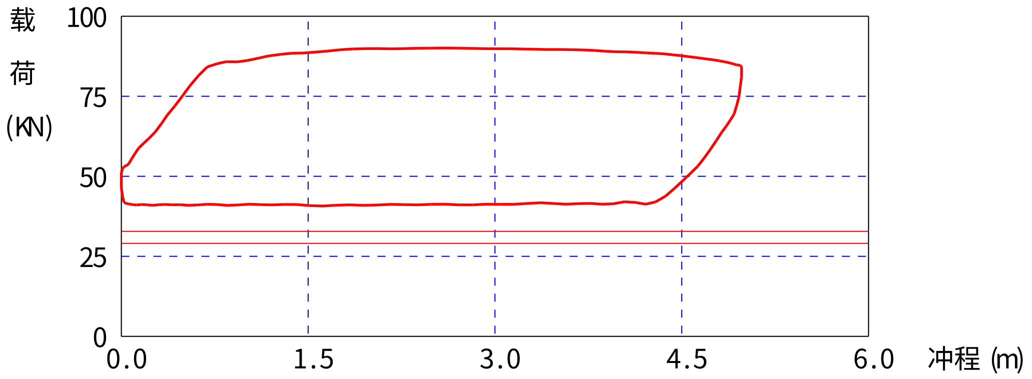

杆 径 二	28	(mm)	液 柱 重	3.75	(KN)	实际产量	0	(t)	上 电 流	0	(A)
杆 长 二	684.65	(m)	杆 柱 重	29.05	(KN)	理论排量	14.78	(t)	下 电 流	0	(A)
杆 径 三	0	(mm)	油 压	0	(MPa)	含 水	0	(%)	动 液 面	181.55	(m)
杆 长 三	0	(m)	套 压	0	(MPa)	泵 效	0	(%)	沉 没 度	520.52	(m)
测 试 人	胡 斌		计 算 人	田 莉 梅		审 核 人	袁 莹 波		单位名称	第一采油厂	

日 期 :2016-12-22 10:21 AM

示 功 图 测 试 报 表

井 号	高 153-443	测试日期	2016年 12月 15日	测试单位	试井队
矿 名	采油七矿	仪器名称	抽油井综合测试仪	分析结果	正常

冲 程	4.91	(m)
冲 次	1.9	(min)
上 载 荷	91.87	(KN)
下 载 荷	41.21	(KN)
泵 径	40	(mm)
泵 深	702.07	(m)
杆 径 一	28	(mm)
杆 长 一	9.14	(m)

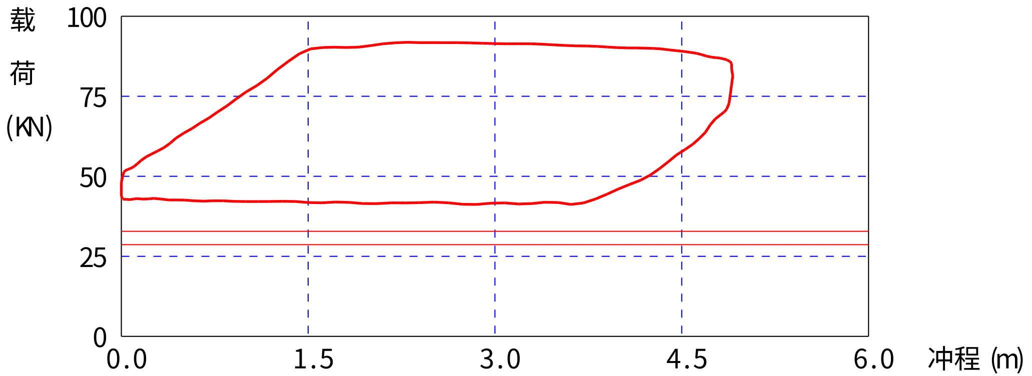

杆 径 二	28	(mm)	液 柱 重	4.17	(KN)	实际产量	6	(t)	上 电 流	95	(A)
杆 长 二	684.65	(m)	杆 柱 重	28.65	(KN)	理论排量	16.44	(t)	下 电 流	84	(A)
杆 径 三	0	(mm)	油 压	0.35	(MPa)	含 水	68.9	(%)	动 液 面	189.47	(m)
杆 长 三	0	(m)	套 压	0.28	(MPa)	泵 效	36.49	(%)	沉 没 度	512.6	(m)
测 试 人	胡 斌		计 算 人	田 莉 梅		审 核 人	袁 莹 波		单位名称	第一采油厂	

日 期 :2016-12-22 10:21 AM

示 功 图 测 试 报 表

井 号	高153-443	测试日期	2016年12月21日	测试单位	五零三队
矿 名	采油七矿	仪器名称	电脑测井仪	分析结果	正常

冲 程	4.88 (m)
冲 次	1.9 (min)
上载荷	97.14 (KN)
下载荷	46.19 (KN)
泵 径	70 (mm)
泵 深	986.47 (m)
杆径一	28 (mm)
杆长一	9.14 (m)

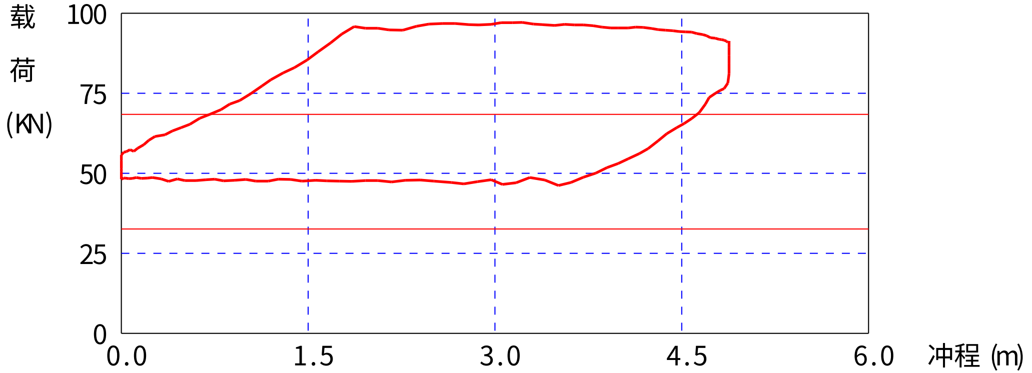

杆径二	25 (mm)	液柱重	35.8 (KN)	实际产量	6.12 (t)	上电流	94 (A)
杆长二	973.44 (m)	杆柱重	32.61 (KN)	理论排量	50.9 (t)	下电流	84 (A)
杆径三	(mm)	油 压	0.33 (MPa)	含 水	75.8 (%)	动液面	-1 (m)
杆长三	0 (m)	套 压	0.26 (MPa)	泵 效	12 (%)	沉没度	0 (m)
测试人	张恕涛	计算人	田莉梅	审核人	袁莹波	单位名称	第一采油厂

日 期 :2016-12-22 10:21 AM

示　功　图　测　试　报　表

井　　号	高 153-443	测试日期	2016年 12月 20日	测试单位	试井队
矿　　名	采油七矿	仪器名称	抽油井综合测试仪	分析结果	正常

冲　　程	4.93	(m)
冲　　次	2	(min)
上 载 荷	90.85	(KN)
下 载 荷	41.76	(KN)
泵　　径	40	(mm)
泵　　深	702.07	(m)
杆 径 一	28	(mm)
杆 长 一	9.14	(m)

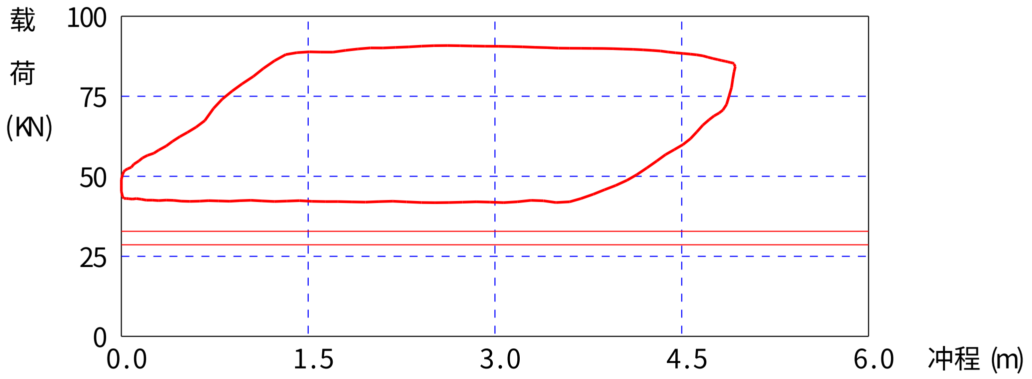

杆 径 二	28	(mm)	液 柱 重	4.21	(KN)	实际产量	6.12	(t)	上 电 流	94	(A)
杆 长 二	684.65	(m)	杆 柱 重	28.61	(KN)	理论排量	16.61	(t)	下 电 流	84	(A)
杆 径 三	0	(mm)	油　　压	0.33	(MPa)	含　　水	75.8	(%)	动 液 面	103.25	(m)
杆 长 三	0	(m)	套　　压	0.26	(MPa)	泵　　效	36.85	(%)	沉 没 度	598.82	(m)
测 试 人	胡　　斌		计 算 人	田 莉 梅		审 核 人	袁 莹 波		单位名称	第一采油厂	

日 期:2016-12-22 10:21 AM
