# Supplementary material for: Diagnosing injection-production system faults in the same well using the rough set-LVQ neural network
Source: PLoS One. 2023 Nov 27;18(11):e0291346. doi: 10.1371/journal.pone.0291346 (PMC10681231; doi:10.1371/journal.pone.0291346)
Supplement: S1 File — (ZIP) [file pone.0291346.s001.zip › A total of 770 dynamometer diagrams for 18 pumping wells/G162-50.pdf]

示 功 图 测 试 报 表

井 号	高 162-50		测试日期	2016年 02月 05日	测试单位	试井队	
矿 名	采油五矿		仪器名称	金时诊断仪	分析结果	供液不足	
冲 程	4.8 (m)	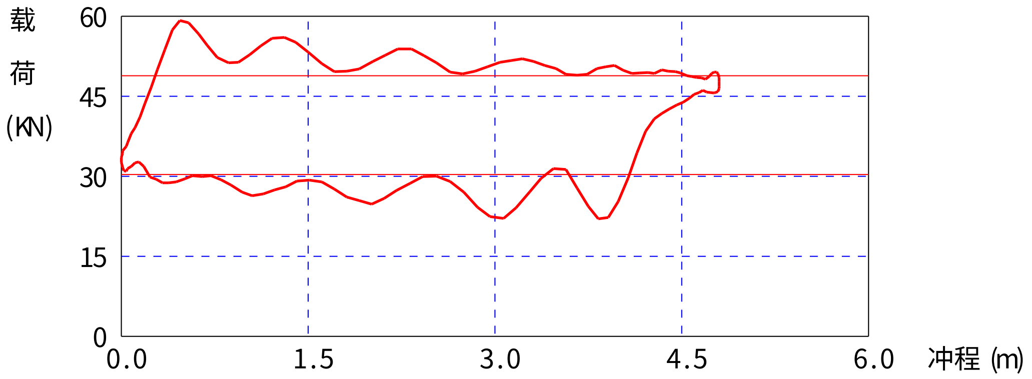					
冲 次	5 (min)						
上 载 荷	59.2 (KN)						
下 载 荷	22.03 (KN)						
泵 径	57 (mm)						
泵 深	927.19 (m)						
杆 径 一	28 (mm)						
杆 长 一	9.14 (m)						
杆 径 二	25 (mm)	液 柱 重	18.51 (KN)	实际产量	66.41 (t)	上 电 流	76 (A)
杆 长 二	907.53 (m)	杆 柱 重	30.34 (KN)	理论排量	88.06 (t)	下 电 流	83 (A)
杆 径 三	22 (mm)	油 压	0.41 (MPa)	含 水	93 (%)	动 液 面	843.68 (m)
杆 长 三	9.14 (m)	套 压	0.42 (MPa)	泵 效	75.41 (%)	沉 没 度	83.51 (m)
测 试 人	李 荣 华	计 算 人	盛 明 波	审 核 人	马 金 江	单位名称	第一采油厂

日 期 :2016-12-22 10:18 AM

示 功 图 测 试 报 表

井 号	高 162-50	测试日期	2016年 03月 11日	测试单位	试井队
矿 名	采油五矿	仪器名称	金时诊断仪	分析结果	供液不足

冲 程	4.82	(m)
冲 次	5.2	(min)
上 载 荷	63.51	(KN)
下 载 荷	22.5	(KN)
泵 径	57	(mm)
泵 深	927.19	(m)
杆 径 一	28	(mm)
杆 长 一	9.14	(m)

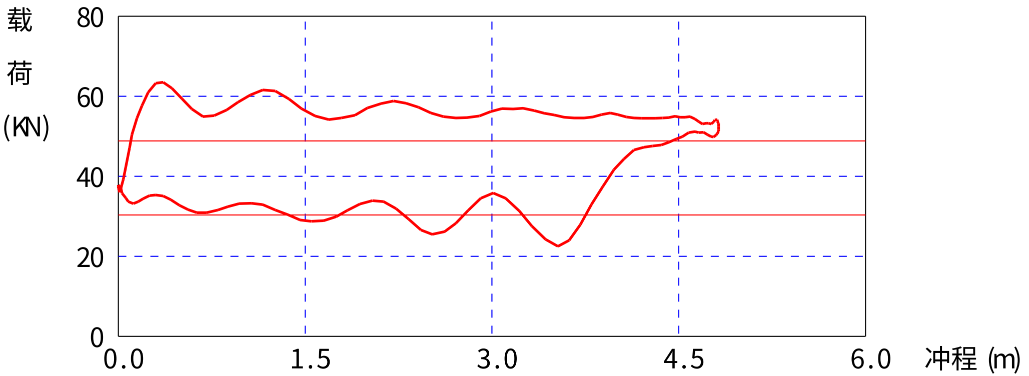

杆 径 二	25 (mm)	液 柱 重	18.51 (KN)	实际产量	66.41 (t)	上 电 流	74 (A)
杆 长 二	907.53 (m)	杆 柱 重	30.34 (KN)	理论排量	90.85 (t)	下 电 流	79 (A)
杆 径 三	22 (mm)	油 压	0.45 (MPa)	含 水	93 (%)	动 液 面	793.33 (m)
杆 长 三	9.14 (m)	套 压	0.47 (MPa)	泵 效	73.1 (%)	沉 没 度	133.86 (m)
测 试 人	李 荣 华	计 算 人	盛 明 波	审 核 人	马 金 江	单位名称	第一采油厂

日 期:2016-12-22 10:18 AM

示 功 图 测 试 报 表

井　　号	高 162-50	测试日期	2016年 12月 13日	测试单位	试井队
矿　　名	采油五矿	仪器名称	抽油井综合测试仪	分析结果	正常

冲　　程	4.79	(m)
冲　　次	2.9	(min)
上 载 荷	100.81	(KN)
下 载 荷	41.53	(KN)
泵　　径	40	(mm)
泵　　深	729.31	(m)
杆 径 一	28	(mm)
杆 长 一	710.49	(m)

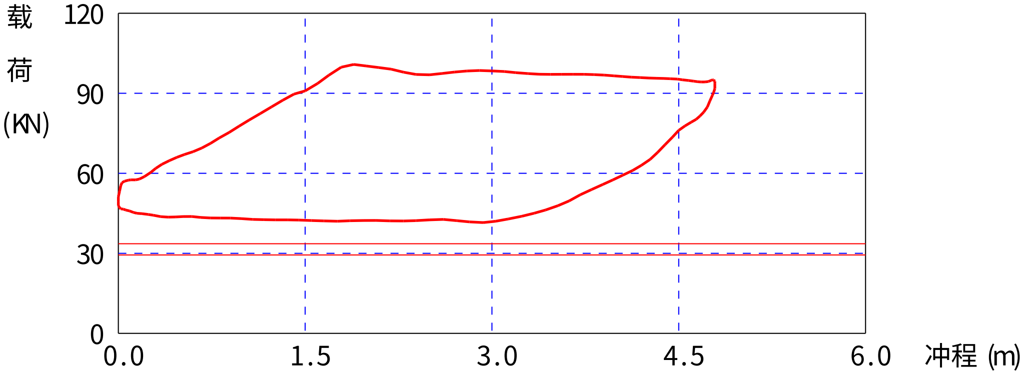

杆 径 二	0	(mm)	液 柱 重	4.21	(KN)	实际产量	11.31	(t)	上 电 流	126	(A)
杆 长 二	0	(m)	杆 柱 重	29.39	(KN)	理论排量	23.73	(t)	下 电 流	74	(A)
杆 径 三	0	(mm)	油　　压	0.45	(MPa)	含　　水	59.9	(%)	动 液 面	169.33	(m)
杆 长 三	0	(m)	套　　压	0.46	(MPa)	泵　　效	47.67	(%)	沉 没 度	559.98	(m)

测 试 人	李 荣 华	计 算 人	盛 明 波	审 核 人	马 金 江	单位名称	第一采油厂

日 期 :2016-12-22 10:18 AM

示 功 图 测 试 报 表

井 号	高 162-50	测试日期	2016年 12月 19日	测试单位	试井队
矿 名	采油五矿	仪器名称	抽油井综合测试仪	分析结果	正常

冲 程	4.75	(m)
冲 次	2.9	(min)
上 载 荷	96.29	(KN)
下 载 荷	39.04	(KN)
泵 径	40	(mm)
泵 深	729.31	(m)
杆 径 一	28	(mm)
杆 长 一	710.49	(m)

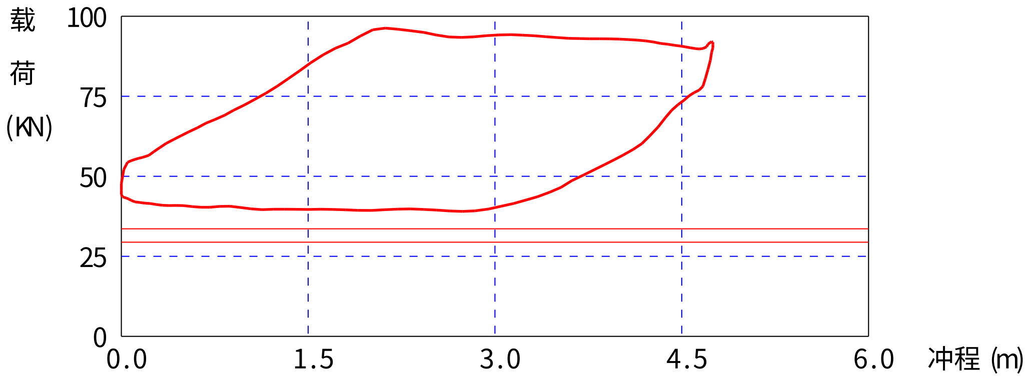

杆 径 二	0	(mm)	液 柱 重	4.18	(KN)	实际产量	10.81	(t)	上 电 流	112	(A)
杆 长 二	0	(m)	杆 柱 重	29.42	(KN)	理论排量	23.35	(t)	下 电 流	78	(A)
杆 径 三	0	(mm)	油 压	0.45	(MPa)	含 水	54.9	(%)	动 液 面	-1	(m)
杆 长 三	0	(m)	套 压	0.46	(MPa)	泵 效	46.29	(%)	沉 没 度	0	(m)
测 试 人	李 荣 华		计 算 人	盛 明 波		审 核 人	马 金 江		单位名称	第一采油厂	

示 功 图 测 试 报 表

井　号	高 162-50	测试日期	2016年 08月 22日	测试单位	试井队
矿　名	采油五矿	仪器名称	抽油井综合测试仪	分析结果	供液不足

冲　程	4.8	(m)
冲　次	4.8	(min)
上 载 荷	63.67	(KN)
下 载 荷	22.34	(KN)
泵　径	57	(mm)
泵　深	927.19	(m)
杆 径 一	28	(mm)
杆 长 一	9.14	(m)

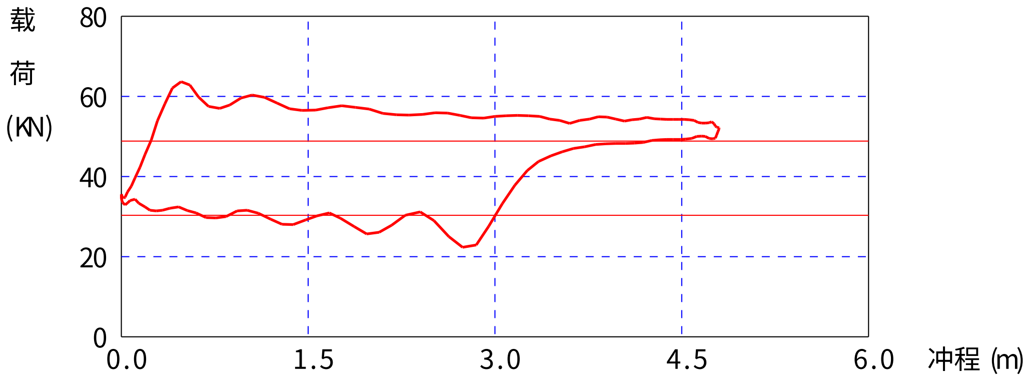

杆 径 二	25	(mm)	液 柱 重	18.52	(KN)	实际产量	78.12	(t)	上 电 流	87	(A)
杆 长 二	907.53	(m)	杆 柱 重	30.33	(KN)	理论排量	83.83	(t)	下 电 流	95	(A)
杆 径 三	22	(mm)	油　压	0.41	(MPa)	含　水	93.4	(%)	动 液 面	800	(m)
杆 长 三	9.14	(m)	套　压	0.45	(MPa)	泵　效	93.19	(%)	沉 没 度	127.19	(m)
测 试 人	李 荣 华		计 算 人	盛 明 波		审 核 人	马 金 江		单位名称	第一采油厂	

示 功 图 测 试 报 表

井 号	高 162-50	测试日期	2016年 09月 18日	测试单位	试井队
矿 名	采油五矿	仪器名称	抽油井综合测试仪	分析结果	正常

冲 程	4.41	(m)
冲 次	2.6	(min)
上 载 荷	97.35	(KN)
下 载 荷	43.04	(KN)
泵 径	57	(mm)
泵 深	927.19	(m)
杆 径 一	28	(mm)
杆 长 一	9.14	(m)

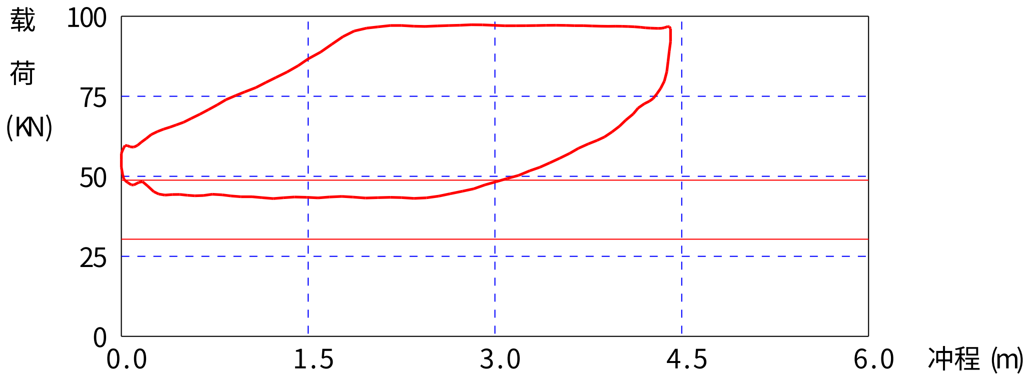

杆 径 二	25	(mm)	液 柱 重	18.44	(KN)	实际产量	9.88	(t)	上 电 流	67	(A)
杆 长 二	907.53	(m)	杆 柱 重	30.35	(KN)	理论排量	41.56	(t)	下 电 流	85	(A)
杆 径 三	22	(mm)	油 压	0.47	(MPa)	含 水	90.3	(%)	动 液 面	130.67	(m)
杆 长 三	9.14	(m)	套 压	0.5	(MPa)	泵 效	23.77	(%)	沉 没 度	796.52	(m)
测 试 人	李 荣 华		计 算 人	盛 明 波		审 核 人	马 金 江		单位名称	第一采油厂	

日 期 :2016-12-22 10:18 AM

示 功 图 测 试 报 表

井　号	高 162-50	测试日期	2016年 10月 11日	测试单位	试井队
矿　名	采油五矿	仪器名称	抽油井综合测试仪	分析结果	正常

冲　程	4.54	(m)
冲　次	2.5	(min)
上载荷	96.06	(KN)
下载荷	37.21	(KN)
泵　径	40	(mm)
泵　深	739.9	(m)
杆径一	28	(mm)
杆长一	9.14	(m)

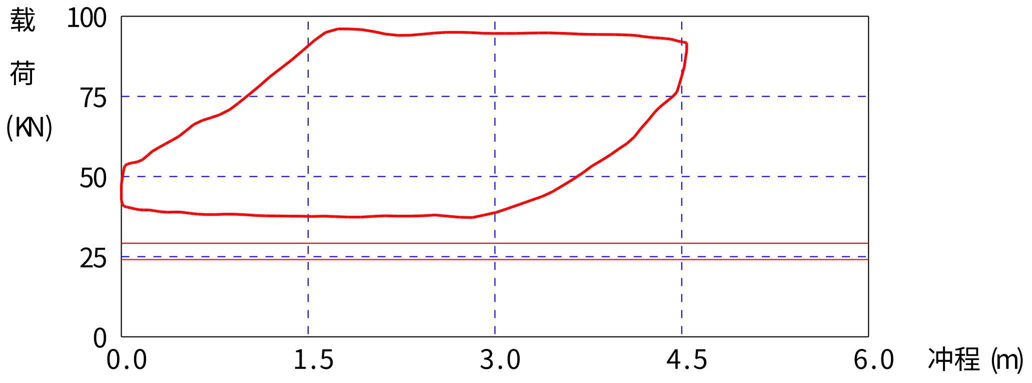

杆径二	25	(mm)	液柱重	5.09	(KN)	实际产量	8.42	(t)	上电流	110	(A)
杆长二	710.49	(m)	杆柱重	24.09	(KN)	理论排量	19.1	(t)	下电流	76	(A)
杆径三	22	(mm)	油　压	0.67	(MPa)	含　水	50	(%)	动液面	196	(m)
杆长三	9.14	(m)	套　压	0.8	(MPa)	泵　效	44.08	(%)	沉没度	543.9	(m)
测试人	李荣华		计算人	盛明波		审核人	马金江		单位名称	第一采油厂	

示 功 图 测 试 报 表

井 号	高 162-50	测试日期	2016年 10月 08日	测试单位	试井队
矿 名	采油五矿	仪器名称	抽油井综合测试仪	分析结果	正常

冲 程	4.52	(m)
冲 次	2.5	(min)
上 载 荷	95.49	(KN)
下 载 荷	37.33	(KN)
泵 径	40	(mm)
泵 深	739.9	(m)
杆 径 一	28	(mm)
杆 长 一	9.14	(m)

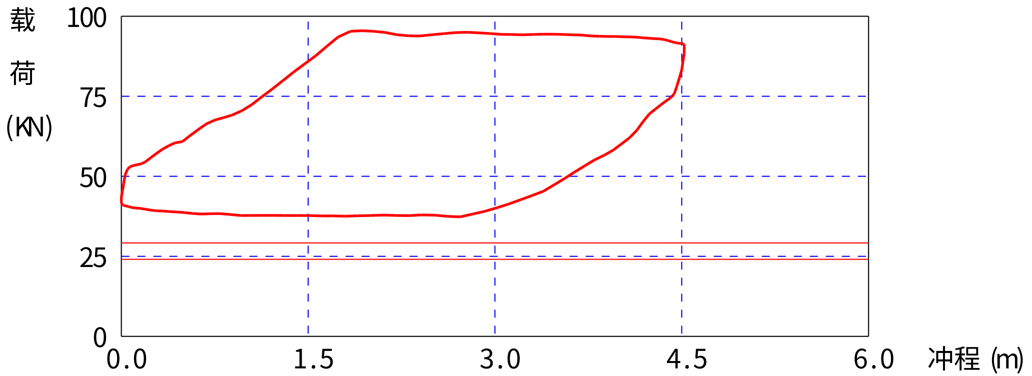

杆 径 二	25	(mm)	液 柱 重	5.12	(KN)	实际产量	9	(t)	上 电 流	110	(A)
杆 长 二	710.49	(m)	杆 柱 重	24.07	(KN)	理论排量	19.13	(t)	下 电 流	73	(A)
杆 径 三	22	(mm)	油 压	0.47	(MPa)	含 水	54	(%)	动 液 面	202.67	(m)
杆 长 三	9.14	(m)	套 压	0.6	(MPa)	泵 效	47.04	(%)	沉 没 度	537.23	(m)
测 试 人	李 荣 华		计 算 人	盛 明 波		审 核 人	马 金 江		单位名称	第一采油厂	

示 功 图 测 试 报 表

井　　号	高 162-50	测试日期	2016年 10月 04日	测试单位	试井队
矿　　名	采油五矿	仪器名称	抽油井综合测试仪	分析结果	正常

冲　　程	4.54	(m)
冲　　次	2.5	(min)
上 载 荷	96.39	(KN)
下 载 荷	37.49	(KN)
泵　　径	40	(mm)
泵　　深	739.9	(m)
杆 径 一	28	(mm)
杆 长 一	9.14	(m)

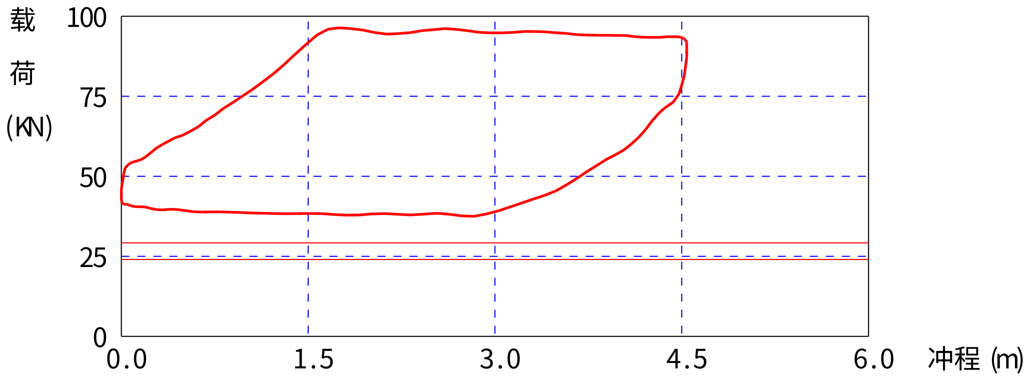

杆 径 二	25	(mm)	液 柱 重	5.2	(KN)	实际产量	9.3	(t)	上 电 流	108	(A)
杆 长 二	710.49	(m)	杆 柱 重	24.02	(KN)	理论排量	19.52	(t)	下 电 流	78	(A)
杆 径 三	22	(mm)	油　　压	0.47	(MPa)	含　　水	64.5	(%)	动 液 面	193.33	(m)
杆 长 三	9.14	(m)	套　　压	0.6	(MPa)	泵　　效	47.65	(%)	沉 没 度	546.57	(m)
测 试 人	李 荣 华		计 算 人	盛 明 波		审 核 人	马 金 江		单位名称	第一采油厂	

日 期 :2016-12-22 10:18 AM

示 功 图 测 试 报 表

井　　号	高162-50		测试日期	2016年10月06日		测试单位	试井队
矿　　名	采油五矿		仪器名称	抽油井综合测试仪		分析结果	正常
冲　　程	4.54 (m)	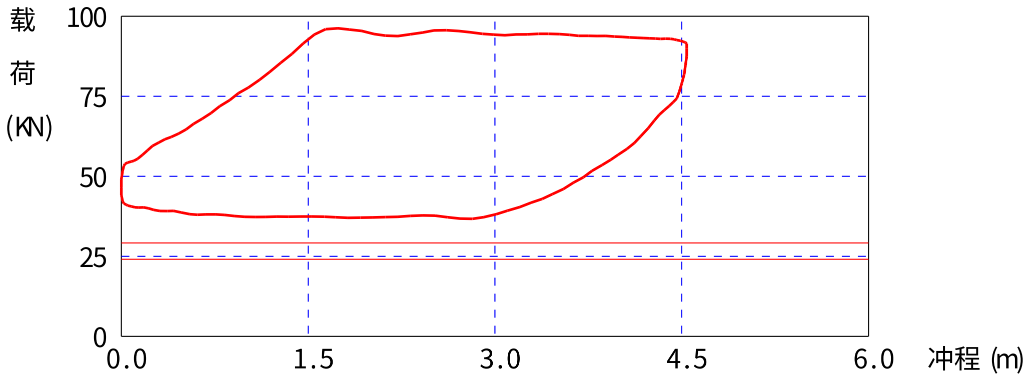					
冲　　次	2.5 (min)						
上 载 荷	96.23 (KN)						
下 载 荷	36.72 (KN)						
泵　　径	40 (mm)						
泵　　深	739.9 (m)						
杆 径 一	28 (mm)						
杆 长 一	9.14 (m)						
杆 径 二	25 (mm)	液 柱 重	5.1 (KN)	实际产量	9.01 (t)	上 电 流	110 (A)
杆 长 二	710.49 (m)	杆 柱 重	24.08 (KN)	理论排量	19.17 (t)	下 电 流	73 (A)
杆 径 三	22 (mm)	油　　压	0.47 (MPa)	含　　水	52.3 (%)	动 液 面	190.86 (m)
杆 长 三	9.14 (m)	套　　压	0.6 (MPa)	泵　　效	47.01 (%)	沉 没 度	549.04 (m)
测 试 人	李 荣 华	计 算 人	盛 明 波	审 核 人	马 金 江	单位名称	第一采油厂

示 功 图 测 试 报 表

井　　号	高 162-50	测试日期	2016年 09月 26日	测试单位	试井队
矿　　名	采油五矿	仪器名称	抽油井综合测试仪	分析结果	正常

冲　　程	4.65	(m)
冲　　次	2.6	(min)
上 载 荷	92.44	(KN)
下 载 荷	38.65	(KN)
泵　　径	57	(mm)
泵　　深	927.19	(m)
杆 径 一	28	(mm)
杆 长 一	9.14	(m)

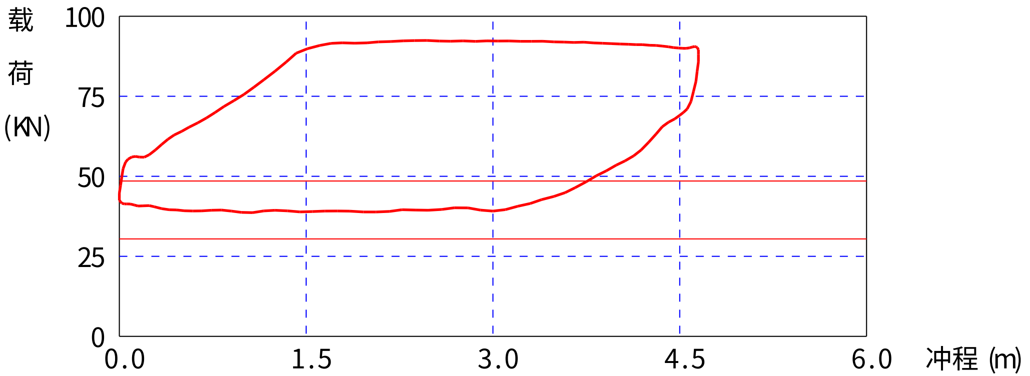

杆 径 二	25	(mm)	液 柱 重	18.09	(KN)	实际产量	10.69	(t)	上 电 流	105	(A)
杆 长 二	907.53	(m)	杆 柱 重	30.44	(KN)	理论排量	42.99	(t)	下 电 流	73	(A)
杆 径 三	22	(mm)	油　　压	0.46	(MPa)	含　　水	77	(%)	动 液 面	44	(m)
杆 长 三	9.14	(m)	套　　压	0.49	(MPa)	泵　　效	24.86	(%)	沉 没 度	883.19	(m)
测 试 人	李 荣 华		计 算 人	盛 明 波		审 核 人	马 金 江		单位名称	第一采油厂	

日 期 :2016-12-22 10:18 AM

示 功 图 测 试 报 表

井 号	高 162-50	测试日期	2016年 10月 31日	测试单位	试井队
矿 名	采油五矿	仪器名称	抽油井综合测试仪	分析结果	正常

冲 程	4.64	(m)
冲 次	2.5	(min)
上 载 荷	98.65	(KN)
下 载 荷	37.75	(KN)
泵 径	40	(mm)
泵 深	729.31	(m)
杆 径 一	28	(mm)
杆 长 一	9.14	(m)

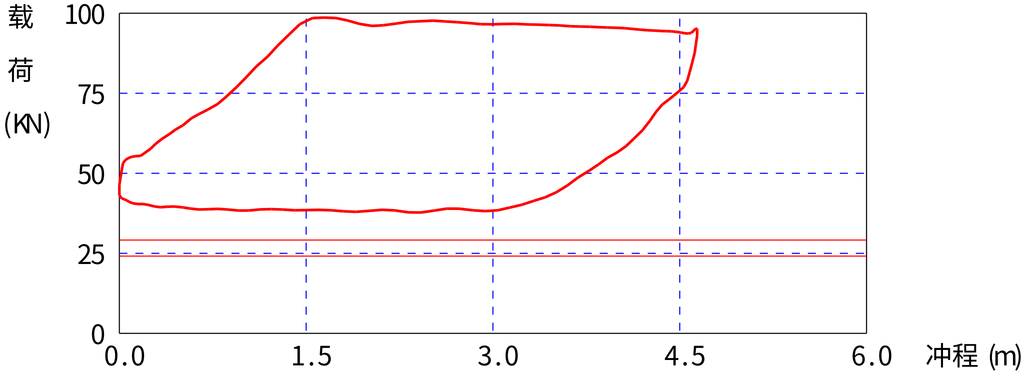

杆 径 二	25	(mm)	液 柱 重	5.02	(KN)	实际产量	5.17	(t)	上 电 流	118	(A)
杆 长 二	710.49	(m)	杆 柱 重	24.13	(KN)	理论排量	19.29	(t)	下 电 流	73	(A)
杆 径 三	22	(mm)	油 压	0.72	(MPa)	含 水	42	(%)	动 液 面	249.33	(m)
杆 长 三	9.14	(m)	套 压	0.78	(MPa)	泵 效	26.81	(%)	沉 没 度	479.98	(m)
测 试 人	李 荣 华		计 算 人	盛 明 波		审 核 人	马 金 江		单位名称	第一采油厂	

示 功 图 测 试 报 表

井 号	高 162-50	测试日期	2016年 11月 02日	测试单位	试井队
矿 名	采油五矿	仪器名称	抽油井综合测试仪	分析结果	正常

冲 程	4.66	(m)
冲 次	2.5	(min)
上 载 荷	100.59	(KN)
下 载 荷	38.74	(KN)
泵 径	40	(mm)
泵 深	729.31	(m)
杆 径 一	28	(mm)
杆 长 一	9.14	(m)

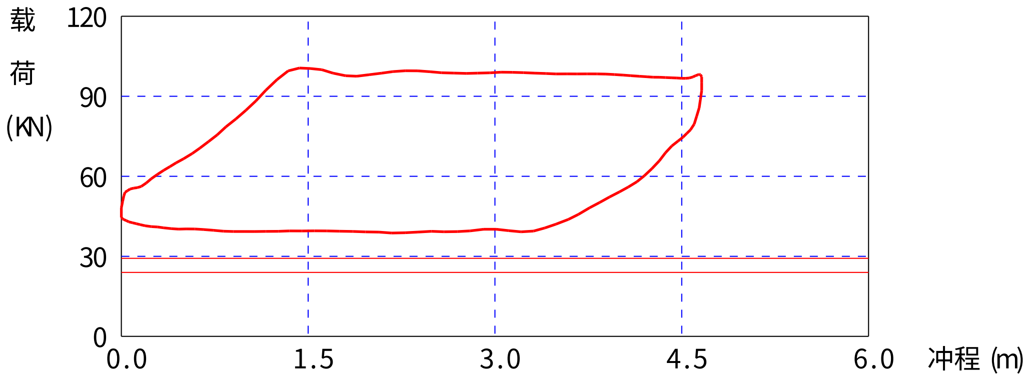

杆 径 二	25	(mm)	液 柱 重	5.28	(KN)	实际产量	1.22	(t)	上 电 流	120	(A)
杆 长 二	710.49	(m)	杆 柱 重	23.97	(KN)	理论排量	20.37	(t)	下 电 流	74	(A)
杆 径 三	22	(mm)	油 压	0.8	(MPa)	含 水	75.8	(%)	动 液 面	323.82	(m)
杆 长 三	9.14	(m)	套 压	0.82	(MPa)	泵 效	5.99	(%)	沉 没 度	405.49	(m)
测 试 人	李 荣 华		计 算 人	盛 明 波		审 核 人	马 金 江		单位名称	第一采油厂	

示 功 图 测 试 报 表

井 号	高 162-50	测试日期	2016年 11月 12日	测试单位	试井队
矿 名	采油五矿	仪器名称	抽油井综合测试仪	分析结果	正常

冲 程	4.83	(m)
冲 次	2.5	(min)
上 载 荷	90.54	(KN)
下 载 荷	36.08	(KN)
泵 径	40	(mm)
泵 深	729.31	(m)
杆 径 一	28	(mm)
杆 长 一	710.49	(m)

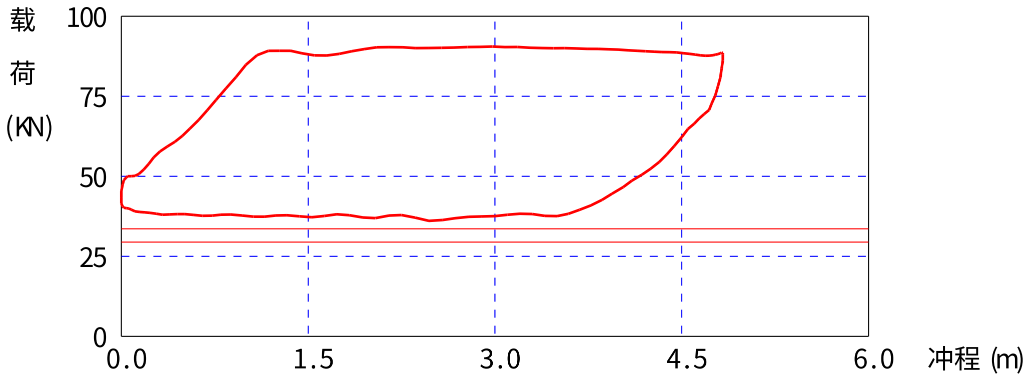

杆 径 二	0	(mm)	液 柱 重	4.15	(KN)	实际产量	4.22	(t)	上 电 流	118	(A)
杆 长 二	0	(m)	杆 柱 重	29.45	(KN)	理论排量	20.32	(t)	下 电 流	73	(A)
杆 径 三	0	(mm)	油 压	0.82	(MPa)	含 水	50	(%)	动 液 面	229.9	(m)
杆 长 三	0	(m)	套 压	0.91	(MPa)	泵 效	20.77	(%)	沉 没 度	499.41	(m)
测 试 人	李 荣 华		计 算 人	盛 明 波		审 核 人	马 金 江		单位名称	第一采油厂	

日 期 :2016-12-22 10:18 AM

示 功 图 测 试 报 表

井 号	高 162-50	测试日期	2016年 11月 03日	测试单位	试井队
矿 名	采油五矿	仪器名称	抽油井综合测试仪	分析结果	正常

冲 程	4.67	(m)
冲 次	2.5	(min)
上 载 荷	100.42	(KN)
下 载 荷	39.59	(KN)
泵 径	40	(mm)
泵 深	729.31	(m)
杆 径 一	28	(mm)
杆 长 一	9.14	(m)

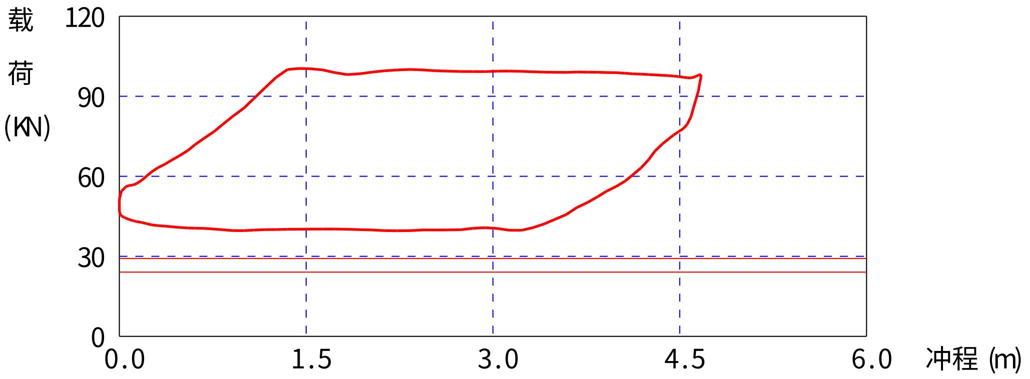

杆 径 二	25	(mm)	液 柱 重	5.11	(KN)	实际产量	4.66	(t)	上 电 流	118	(A)
杆 长 二	710.49	(m)	杆 柱 重	24.08	(KN)	理论排量	19.74	(t)	下 电 流	71	(A)
杆 径 三	22	(mm)	油 压	0.8	(MPa)	含 水	53	(%)	动 液 面	341.33	(m)
杆 长 三	9.14	(m)	套 压	0.82	(MPa)	泵 效	23.61	(%)	沉 没 度	387.98	(m)
测 试 人	李 荣 华		计 算 人	盛 明 波		审 核 人	马 金 江		单位名称	第一采油厂	

日 期 :2016-12-22 10:18 AM

示　功　图　测　试　报　表

井　　号	高 162-50	测试日期	2016年 11月 23日	测试单位	试井队
矿　　名	采油五矿	仪器名称	抽油井综合测试仪	分析结果	正常

冲　　程	4.77	(m)
冲　　次	2.5	(min)
上 载 荷	96.26	(KN)
下 载 荷	39.47	(KN)
泵　　径	40	(mm)
泵　　深	729.31	(m)
杆 径 一	28	(mm)
杆 长 一	710.49	(m)

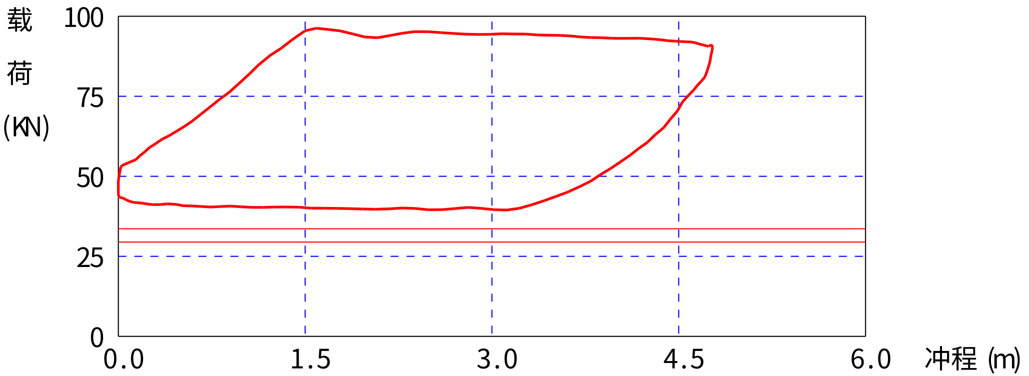

杆 径 二	0	(mm)	液 柱 重	4.15	(KN)	实际产量	6.2	(t)	上 电 流	119	(A)
杆 长 二	0	(m)	杆 柱 重	29.46	(KN)	理论排量	20.05	(t)	下 电 流	75	(A)
杆 径 三	0	(mm)	油　　压	0.62	(MPa)	含　　水	49.3	(%)	动 液 面	166.15	(m)
杆 长 三	0	(m)	套　　压	0.71	(MPa)	泵　　效	30.93	(%)	沉 没 度	563.16	(m)
测 试 人	李 荣 华		计 算 人	盛 明 波		审 核 人	马 金 江		单位名称	第一采油厂	

日 期 :2016-12-22 10:18 AM

示 功 图 测 试 报 表

井　号	高162-50	测试日期	2016年11月30日	测试单位	试井队
矿　名	采油五矿	仪器名称	抽油井综合测试仪	分析结果	正常

冲　程	4.74	(m)
冲　次	2.9	(min)
上载荷	100.84	(KN)
下载荷	39.21	(KN)
泵　径	40	(mm)
泵　深	729.31	(m)
杆径一	28	(mm)
杆长一	710.49	(m)

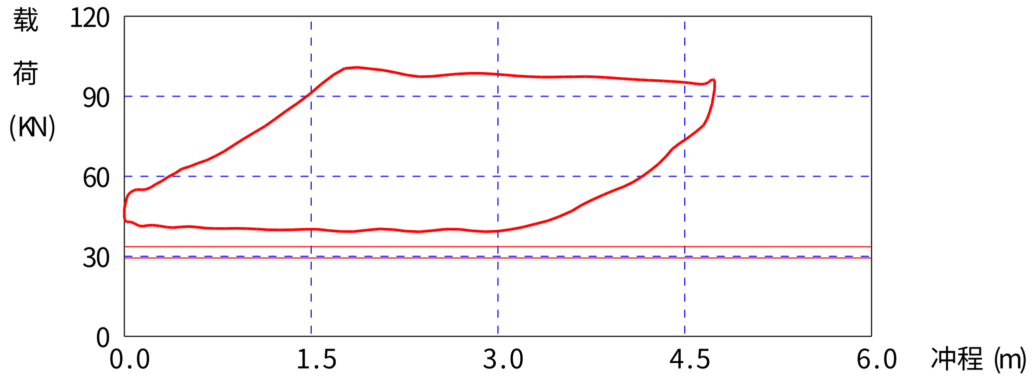

杆径二	0	(mm)	液柱重	4.22	(KN)	实际产量	7.4	(t)	上电流	118	(A)
杆长二	0	(m)	杆柱重	29.39	(KN)	理论排量	23.52	(t)	下电流	73	(A)
杆径三	0	(mm)	油　压	0.62	(MPa)	含　水	61	(%)	动液面	244	(m)
杆长三	0	(m)	套　压	0.71	(MPa)	泵　效	31.47	(%)	沉没度	485.31	(m)
测试人	李荣华		计算人	盛明波		审核人	马金江		单位名称	第一采油厂	

日 期 :2016-12-22 10:18 AM

示 功 图 测 试 报 表

井　　号	高 162-50	测试日期	2016年 11月 22日	测试单位	试井队
矿　　名	采油五矿	仪器名称	抽油井综合测试仪	分析结果	正常

冲　　程	4.74	(m)
冲　　次	2.5	(min)
上 载 荷	97.47	(KN)
下 载 荷	37.31	(KN)
泵　　径	40	(mm)
泵　　深	729.31	(m)
杆 径 一	28	(mm)
杆 长 一	710.49	(m)

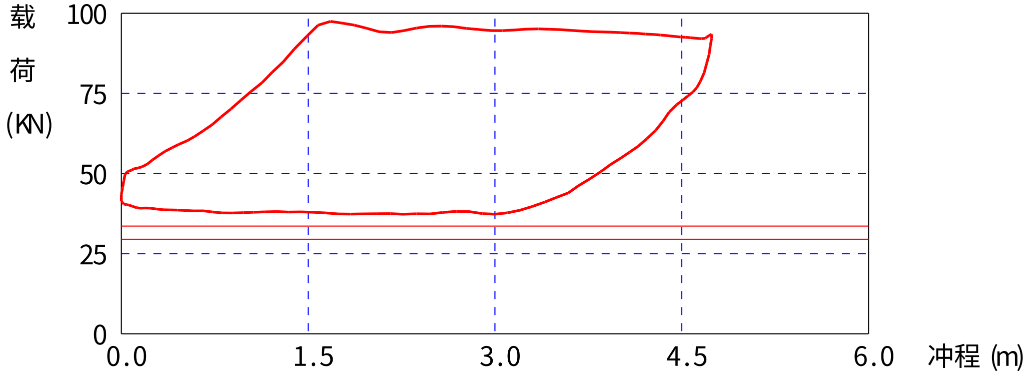

杆 径 二	0	(mm)	液 柱 重	4.11	(KN)	实际产量	6.08	(t)	上 电 流	119	(A)
杆 长 二	0	(m)	杆 柱 重	29.49	(KN)	理论排量	19.76	(t)	下 电 流	76	(A)
杆 径 三	0	(mm)	油　　压	0.62	(MPa)	含　　水	44	(%)	动 液 面	143.52	(m)
杆 长 三	0	(m)	套　　压	0.71	(MPa)	泵　　效	30.77	(%)	沉 没 度	585.79	(m)
测 试 人	李 荣 华		计 算 人	盛 明 波		审 核 人	马 金 江		单位名称	第一采油厂	

日 期 :2016-12-22 10:18 AM

示 功 图 测 试 报 表

井 号	高162-50	测试日期	2016年11月28日	测试单位	试井队
矿 名	采油五矿	仪器名称	抽油井综合测试仪	分析结果	正常

冲 程	4.72	(m)
冲 次	2.5	(min)
上 载 荷	95.5	(KN)
下 载 荷	37.19	(KN)
泵 径	40	(mm)
泵 深	729.31	(m)
杆 径 一	28	(mm)
杆 长 一	710.49	(m)

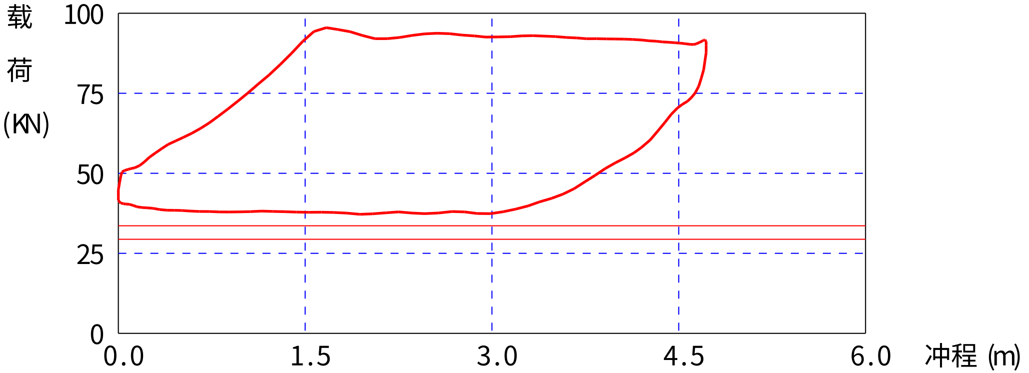

杆 径 二	0	(mm)	液 柱 重	4.22	(KN)	实际产量	6.9	(t)	上 电 流	120	(A)
杆 长 二	0	(m)	杆 柱 重	29.39	(KN)	理论排量	20.19	(t)	下 电 流	76	(A)
杆 径 三	0	(mm)	油 压	0.62	(MPa)	含 水	61.1	(%)	动 液 面	212	(m)
杆 长 三	0	(m)	套 压	0.71	(MPa)	泵 效	34.18	(%)	沉 没 度	517.31	(m)
测 试 人	李 荣 华		计 算 人	盛 明 波		审 核 人	马 金 江		单位名称	第一采油厂	

日 期 :2016-12-22 10:18 AM

示 功 图 测 试 报 表

井 号	高 162-50	测试日期	2016年 11月 24日	测试单位	试井队
矿 名	采油五矿	仪器名称	抽油井综合测试仪	分析结果	正常

冲 程	4.74	(m)
冲 次	2.5	(min)
上 载 荷	96.31	(KN)
下 载 荷	37.26	(KN)
泵 径	40	(mm)
泵 深	729.31	(m)
杆 径 一	28	(mm)
杆 长 一	710.49	(m)

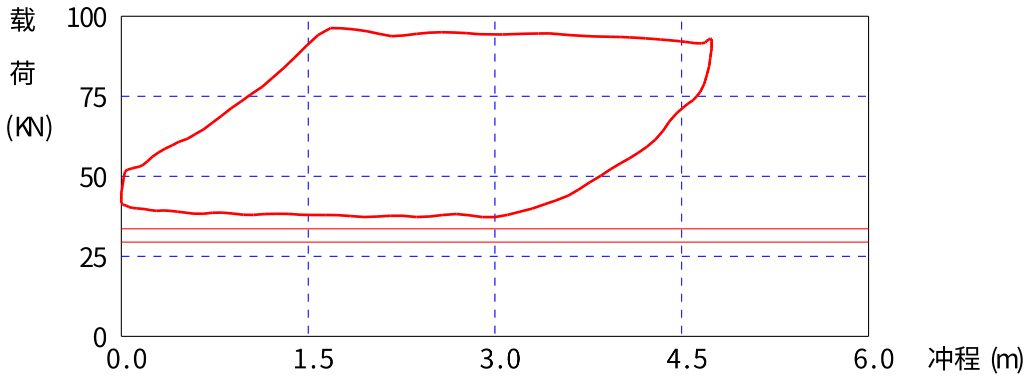

杆 径 二	0	(mm)	液 柱 重	4.16	(KN)	实际产量	6.5	(t)	上 电 流	116	(A)
杆 长 二	0	(m)	杆 柱 重	29.44	(KN)	理论排量	20.01	(t)	下 电 流	74	(A)
杆 径 三	0	(mm)	油 压	0.62	(MPa)	含 水	52.3	(%)	动 液 面	157.33	(m)
杆 长 三	0	(m)	套 压	0.71	(MPa)	泵 效	32.48	(%)	沉 没 度	571.98	(m)
测 试 人	李 荣 华		计 算 人	盛 明 波		审 核 人	马 金 江		单位名称	第一采油厂	

示 功 图 测 试 报 表

井 号	高 162-50	测试日期	2016年 11月 26日	测试单位	试井队
矿 名	采油五矿	仪器名称	抽油井综合测试仪	分析结果	正常

冲 程	4.74	(m)
冲 次	2.5	(min)
上 载 荷	95.29	(KN)
下 载 荷	37.81	(KN)
泵 径	40	(mm)
泵 深	729.31	(m)
杆 径 一	28	(mm)
杆 长 一	710.49	(m)

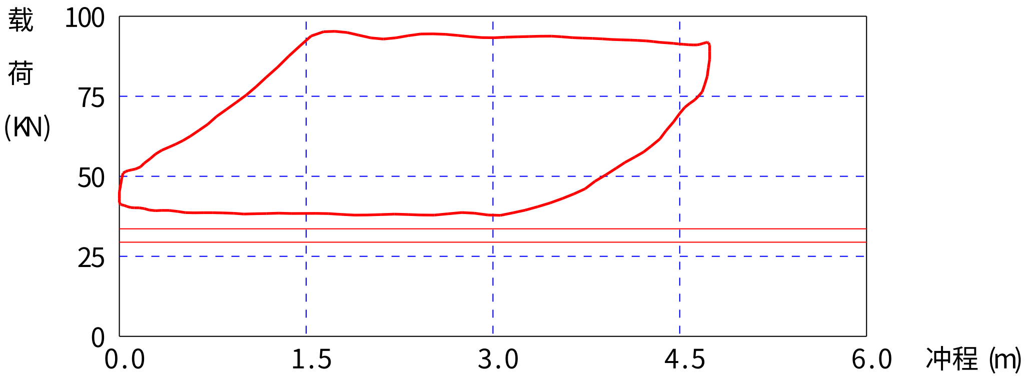

杆 径 二	0	(mm)	液 柱 重	4.18	(KN)	实际产量	6.9	(t)	上 电 流	120	(A)
杆 长 二	0	(m)	杆 柱 重	29.42	(KN)	理论排量	20.1	(t)	下 电 流	77	(A)
杆 径 三	0	(mm)	油 压	0.62	(MPa)	含 水	55.1	(%)	动 液 面	175.39	(m)
杆 长 三	0	(m)	套 压	0.71	(MPa)	泵 效	34.34	(%)	沉 没 度	553.92	(m)
测 试 人	李 荣 华		计 算 人	盛 明 波		审 核 人	马 金 江		单位名称	第一采油厂	

示 功 图 测 试 报 表

井 号	高 162-50	测试日期	2016年 12月 08日	测试单位	试井队
矿 名	采油五矿	仪器名称	抽油井综合测试仪	分析结果	正常

冲 程	4.75	(m)
冲 次	2.9	(min)
上 载 荷	99.18	(KN)
下 载 荷	38.24	(KN)
泵 径	40	(mm)
泵 深	729.31	(m)
杆 径 一	28	(mm)
杆 长 一	710.49	(m)

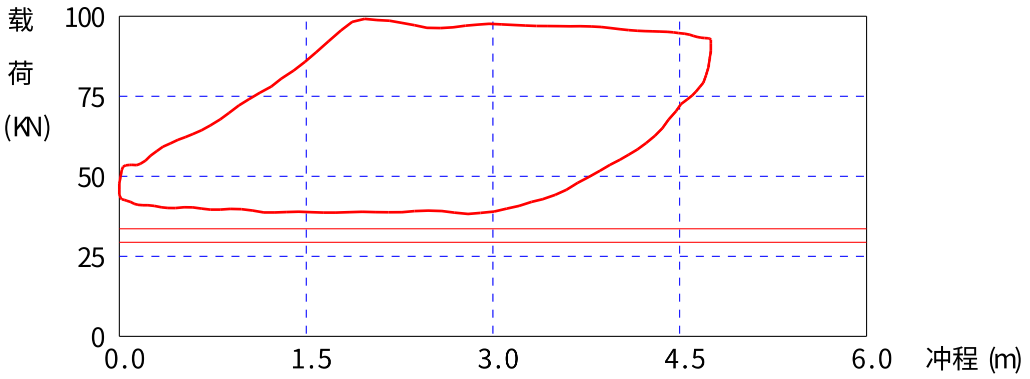

杆 径 二	0	(mm)	液 柱 重	4.23	(KN)	实际产量	11.62	(t)	上 电 流	117	(A)
杆 长 二	0	(m)	杆 柱 重	29.38	(KN)	理论排量	23.62	(t)	下 电 流	73	(A)
杆 径 三	0	(mm)	油 压	0.42	(MPa)	含 水	62.5	(%)	动 液 面	186.67	(m)
杆 长 三	0	(m)	套 压	0.51	(MPa)	泵 效	49.2	(%)	沉 没 度	542.64	(m)
测 试 人	李 荣 华		计 算 人	盛 明 波		审 核 人	马 金 江		单位名称	第一采油厂	

示 功 图 测 试 报 表

井　号	高 162-50	测试日期	2016年 01月 07日	测试单位	试井队
矿　名	采油五矿	仪器名称	金时诊断仪	分析结果	供液不足

冲　程	4.89	(m)
冲　次	5.1	(min)
上 载 荷	58.7	(KN)
下 载 荷	22.03	(KN)
泵　径	57	(mm)
泵　深	927.19	(m)
杆 径 一	28	(mm)
杆 长 一	9.14	(m)

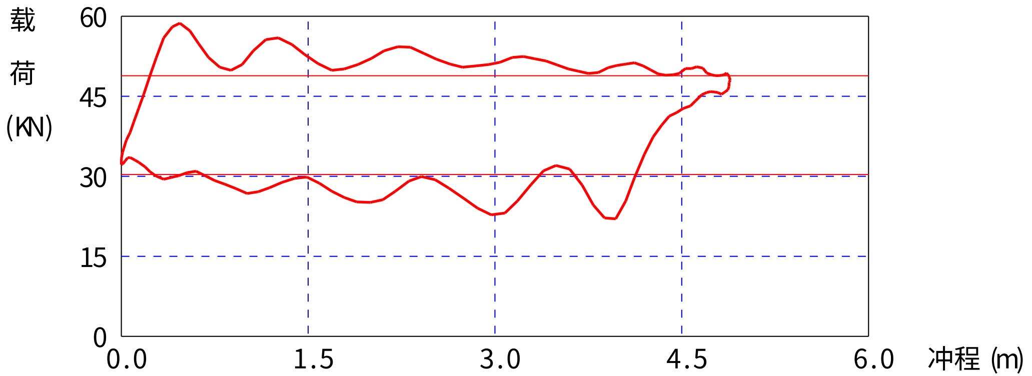

杆 径 二	25 (mm)	液 柱 重	18.51 (KN)	实际产量	63.68 (t)	上 电 流	75 (A)
杆 长 二	907.53 (m)	杆 柱 重	30.34 (KN)	理论排量	90.49 (t)	下 电 流	80 (A)
杆 径 三	22 (mm)	油　压	0.52 (MPa)	含　水	93 (%)	动 液 面	836.38 (m)
杆 长 三	9.14 (m)	套　压	0.55 (MPa)	泵　效	70.37 (%)	沉 没 度	90.81 (m)
测 试 人	李 荣 华	计 算 人	盛 明 波	审 核 人	马 金 江	单位名称	第一采油厂

日 期 :2016-12-22 10:18 AM

示 功 图 测 试 报 表

井 号	高 162-50	测试日期	2016年 04月 08日	测试单位	试井队
矿 名	采油五矿	仪器名称	金时诊断仪	分析结果	供液不足

冲 程	4.82	(m)
冲 次	5.5	(min)
上 载 荷	56.12	(KN)
下 载 荷	22.45	(KN)
泵 径	57	(mm)
泵 深	927.19	(m)
杆 径 一	28	(mm)
杆 长 一	9.14	(m)

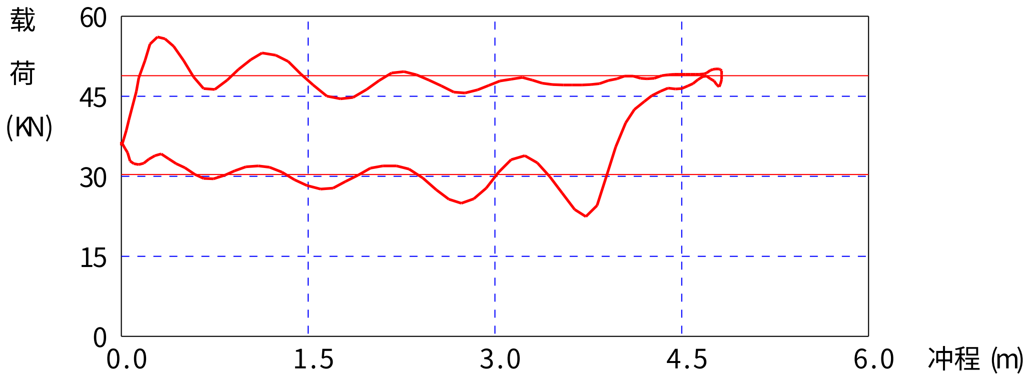

杆 径 二	25 (mm)	液 柱 重	18.52 (KN)	实际产量	60.29 (t)	上 电 流	76 (A)
杆 长 二	907.53 (m)	杆 柱 重	30.34 (KN)	理论排量	96.98 (t)	下 电 流	76 (A)
杆 径 三	22 (mm)	油 压	0.4 (MPa)	含 水	93.3 (%)	动 液 面	893.33 (m)
杆 长 三	9.14 (m)	套 压	0.42 (MPa)	泵 效	62.16 (%)	沉 没 度	33.86 (m)
测 试 人	李 荣 华	计 算 人	盛 明 波	审 核 人	马 金 江	单位名称	第一采油厂

日 期 :2016-12-22 10:18 AM

示 功 图 测 试 报 表

井　号	高 162-50	测试日期	2016年 05月 09日	测试单位	试井队
矿　名	采油五矿	仪器名称	抽油井综合测试仪	分析结果	供液不足

冲　程	4.8 (m)
冲　次	5 (min)
上载荷	58.02 (KN)
下载荷	21.85 (KN)
泵　径	57 (mm)
泵　深	927.19 (m)
杆径一	28 (mm)
杆长一	9.14 (m)

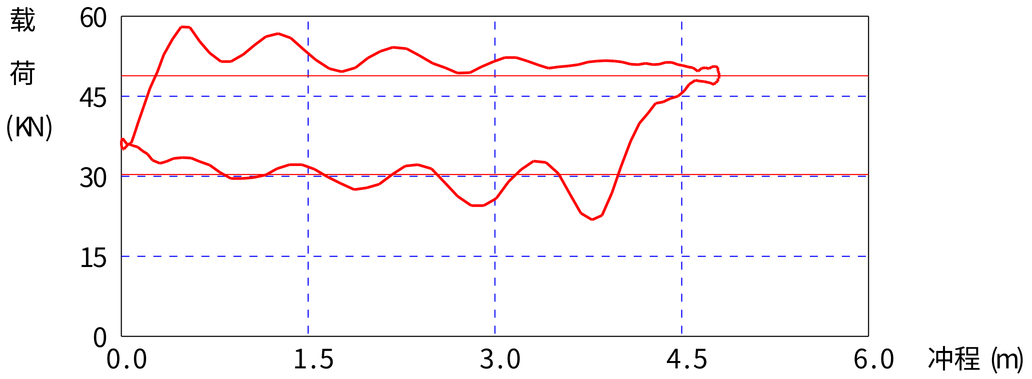

杆径二	25 (mm)	液柱重	18.51 (KN)	实际产量	65.01 (t)	上电流	74 (A)
杆长二	907.53 (m)	杆柱重	30.34 (KN)	理论排量	86.8 (t)	下电流	78 (A)
杆径三	22 (mm)	油　压	0.51 (MPa)	含　水	93 (%)	动液面	898.84 (m)
杆长三	9.14 (m)	套　压	0.6 (MPa)	泵　效	74.9 (%)	沉没度	28.35 (m)
测试人	李荣华	计算人	盛明波	审核人	马金江	单位名称	第一采油厂

日 期 :2016-12-22 10:18 AM

示 功 图 测 试 报 表

井　　号	高 162-50	测试日期	2016年 06月 06日	测试单位	试井队
矿　　名	采油五矿	仪器名称	抽油井综合测试仪	分析结果	供液不足

冲　　程	4.8	(m)
冲　　次	5.5	(min)
上 载 荷	59.1	(KN)
下 载 荷	22.94	(KN)
泵　　径	57	(mm)
泵　　深	927.19	(m)
杆 径 一	28	(mm)
杆 长 一	9.14	(m)

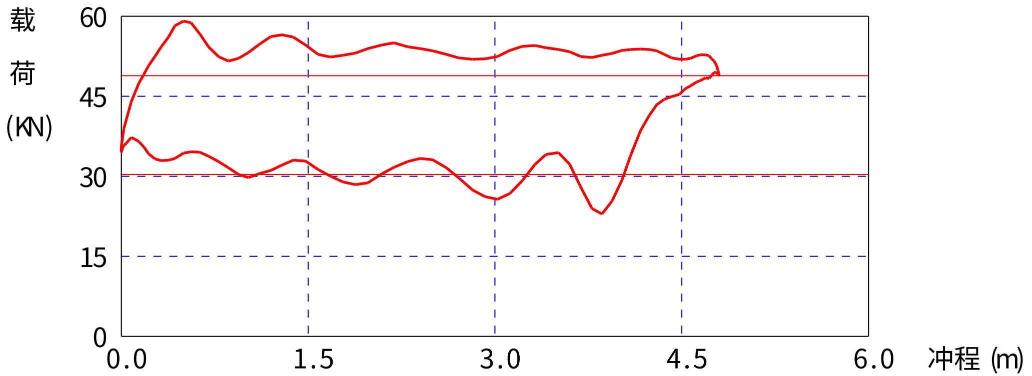

杆 径 二	25	(mm)	液 柱 重	18.52	(KN)	实际产量	66.88	(t)	上 电 流	82	(A)
杆 长 二	907.53	(m)	杆 柱 重	30.34	(KN)	理论排量	96.1	(t)	下 电 流	82	(A)
杆 径 三	22	(mm)	油　　压	0.54	(MPa)	含　　水	93.3	(%)	动 液 面	872.1	(m)
杆 长 三	9.14	(m)	套　　压	0.65	(MPa)	泵　　效	69.6	(%)	沉 没 度	55.09	(m)
测 试 人	李 荣 华		计 算 人	盛 明 波		审 核 人	马 金 江		单位名称	第一采油厂	

示 功 图 测 试 报 表

井　号	高 162-50	测试日期	2016年 07月 04日	测试单位	试井队
矿　名	采油五矿	仪器名称	抽油井综合测试仪	分析结果	供液不足

冲　程	4.8	(m)
冲　次	5.5	(min)
上 载 荷	54.98	(KN)
下 载 荷	21.53	(KN)
泵　径	57	(mm)
泵　深	927.19	(m)
杆 径 一	28	(mm)
杆 长 一	9.14	(m)

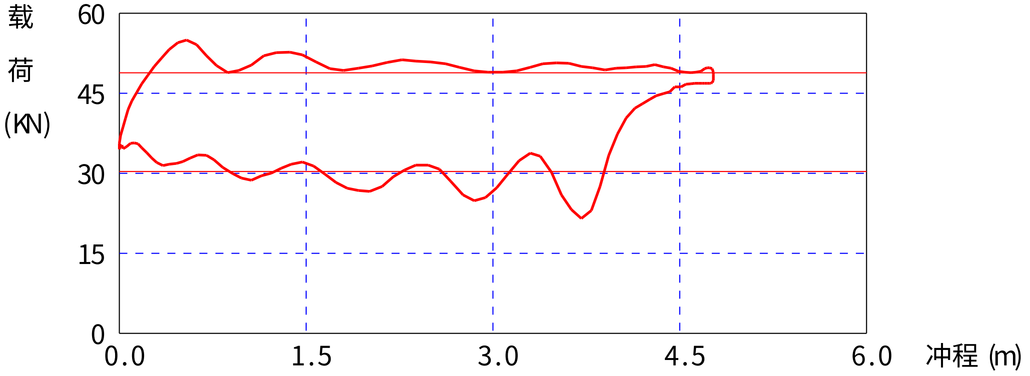

杆 径 二	25	(mm)	液 柱 重	18.5	(KN)	实际产量	67.37	(t)	上 电 流	80	(A)
杆 长 二	907.53	(m)	杆 柱 重	30.34	(KN)	理论排量	95.99	(t)	下 电 流	78	(A)
杆 径 三	22	(mm)	油　压	0.58	(MPa)	含　水	92.5	(%)	动 液 面	867.74	(m)
杆 长 三	9.14	(m)	套　压	0.6	(MPa)	泵　效	70.19	(%)	沉 没 度	59.45	(m)
测 试 人	李 荣 华		计 算 人	盛 明 波		审 核 人	马 金 江		单位名称	第一采油厂	

日 期 :2016-12-22 10:18 AM

示 功 图 测 试 报 表

井 号	高 162-50	测试日期	2016年 10月 02日	测试单位	试井队
矿 名	采油五矿	仪器名称	抽油井综合测试仪	分析结果	正常

冲 程	4.51	(m)
冲 次	2.5	(min)
上 载 荷	97.14	(KN)
下 载 荷	38.92	(KN)
泵 径	40	(mm)
泵 深	739.9	(m)
杆 径 一	28	(mm)
杆 长 一	9.14	(m)

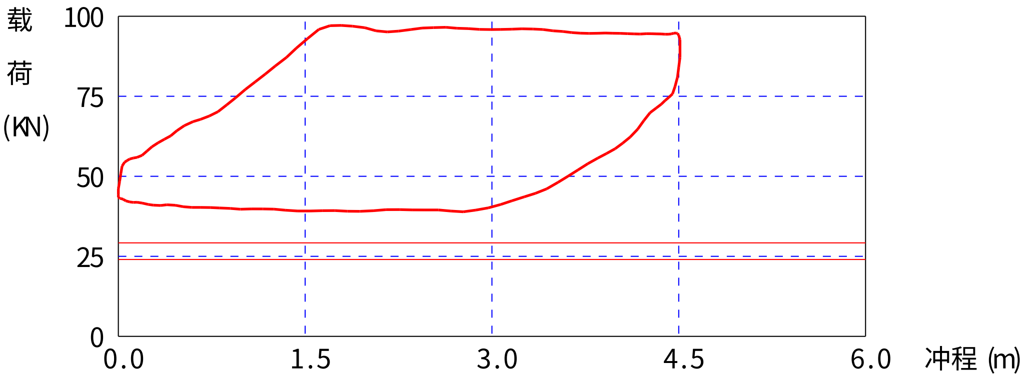

杆 径 二	25	(mm)	液 柱 重	5.19	(KN)	实际产量	9.64	(t)	上 电 流	108	(A)
杆 长 二	710.49	(m)	杆 柱 重	24.02	(KN)	理论排量	19.38	(t)	下 电 流	79	(A)
杆 径 三	22	(mm)	油 压	0.47	(MPa)	含 水	64.2	(%)	动 液 面	190.67	(m)
杆 长 三	9.14	(m)	套 压	0.6	(MPa)	泵 效	49.74	(%)	沉 没 度	549.23	(m)
测 试 人	李 荣 华		计 算 人	盛 明 波		审 核 人	马 金 江		单位名称	第一采油厂	

日 期 :2016-12-22 10:18 AM

示　功　图　测　试　报　表

井　号	高 162-50	测试日期	2016年 09月 14日	测试单位	试井队
矿　名	采油五矿	仪器名称	抽油井综合测试仪	分析结果	其它

冲　程	4.53	(m)
冲　次	1.6	(min)
上载荷	96.67	(KN)
下载荷	38.92	(KN)
泵　径	57	(mm)
泵　深	927.19	(m)
杆径一	28	(mm)
杆长一	9.14	(m)

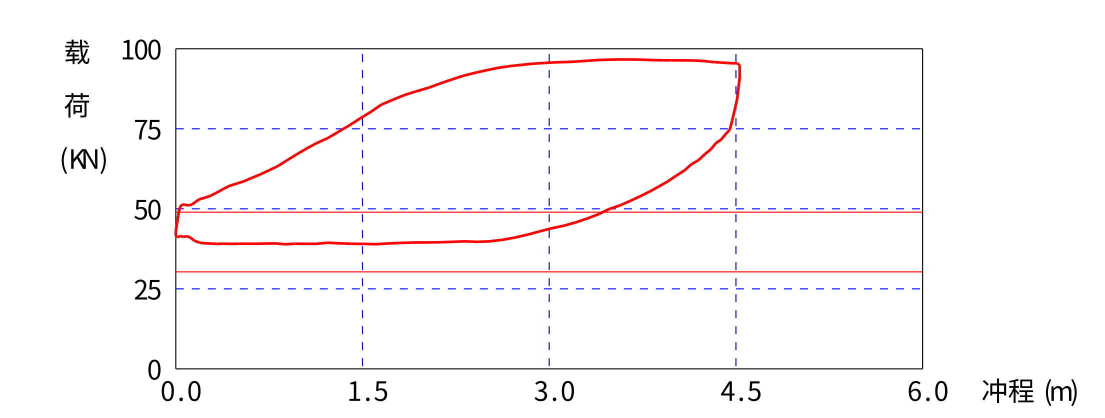

杆径二	25	(mm)	液柱重	18.63	(KN)	实际产量	21.03	(t)	上电流	127	(A)
杆长二	907.53	(m)	杆柱重	30.31	(KN)	理论排量	26.54	(t)	下电流	85	(A)
杆径三	22	(mm)	油　压	0.48	(MPa)	含　水	97.5	(%)	动液面	140	(m)
杆长三	9.14	(m)	套　压	0.52	(MPa)	泵　效	79.24	(%)	沉没度	787.19	(m)
测试人	李荣华		计算人	盛明波		审核人	马金江		单位名称	第一采油厂	

示　功　图　测　试　报　表

井　　号	高 162-50	测试日期	2016年 09月 23日	测试单位	试井队
矿　　名	采油五矿	仪器名称	抽油井综合测试仪	分析结果	正常

冲　　程	4.54	(m)
冲　　次	2.5	(min)
上 载 荷	96.38	(KN)
下 载 荷	37.53	(KN)
泵　　径	57	(mm)
泵　　深	927.19	(m)
杆 径 一	28	(mm)
杆 长 一	9.14	(m)

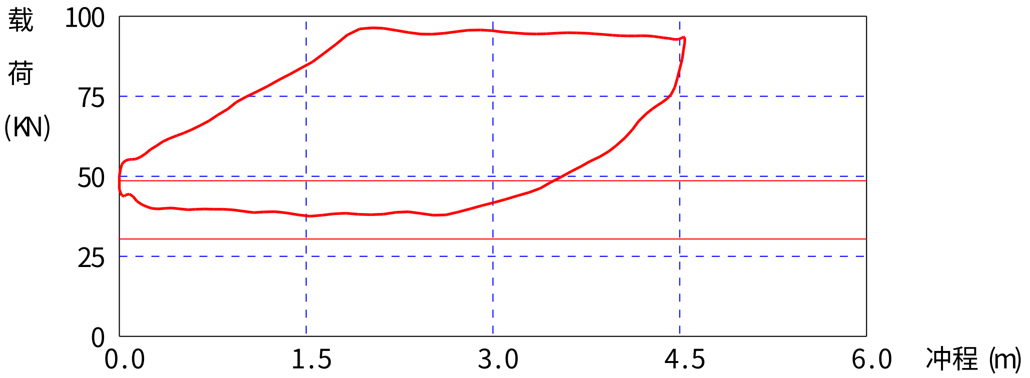

杆 径 二	25	(mm)	液 柱 重	18.19	(KN)	实际产量	11.07	(t)	上 电 流	99	(A)
杆 长 二	907.53	(m)	杆 柱 重	30.42	(KN)	理论排量	40.57	(t)	下 电 流	71	(A)
杆 径 三	22	(mm)	油　　压	0.46	(MPa)	含　　水	80.5	(%)	动 液 面	-1	(m)
杆 长 三	9.14	(m)	套　　压	0.49	(MPa)	泵　　效	27.29	(%)	沉 没 度	0	(m)
测 试 人	李 荣 华		计 算 人	盛 明 波		审 核 人	马 金 江		单位名称	第一采油厂	

日 期 :2016-12-22 10:18 AM

示 功 图 测 试 报 表

井　　号	高 162-50	测试日期	2016年 10月 13日	测试单位	试井队
矿　　名	采油五矿	仪器名称	抽油井综合测试仪	分析结果	正常

冲　　程	4.66	(m)
冲　　次	2.5	(min)
上 载 荷	102.46	(KN)
下 载 荷	40.1	(KN)
泵　　径	40	(mm)
泵　　深	739.9	(m)
杆 径 一	28	(mm)
杆 长 一	9.14	(m)

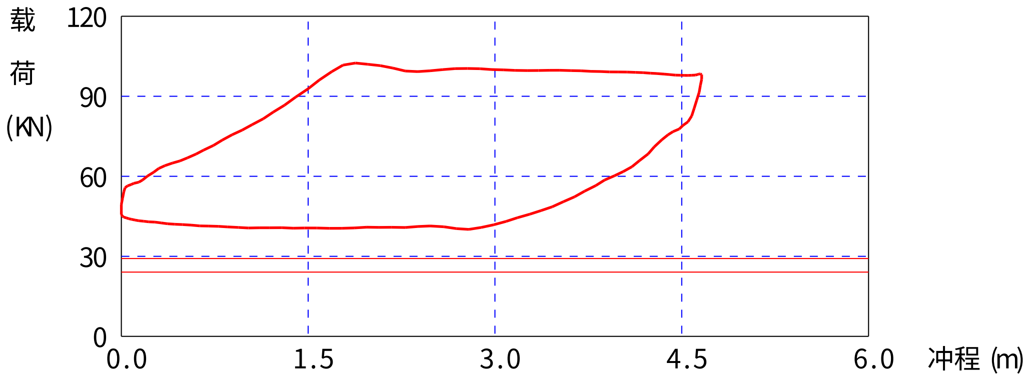

杆 径 二	25 (mm)	液 柱 重	5.09 (KN)	实际产量	7.49 (t)	上 电 流	111 (A)
杆 长 二	710.49 (m)	杆 柱 重	24.09 (KN)	理论排量	19.62 (t)	下 电 流	72 (A)
杆 径 三	22 (mm)	油　　压	0.67 (MPa)	含　　水	50.5 (%)	动 液 面	199.26 (m)
杆 长 三	9.14 (m)	套　　压	0.8 (MPa)	泵　　效	38.17 (%)	沉 没 度	540.64 (m)
测 试 人	李 荣 华	计 算 人	盛 明 波	审 核 人	马 金 江	单位名称	第一采油厂

日 期 :2016-12-22 10:18 AM

示 功 图 测 试 报 表

井 号	高 162-50		测试日期	2016年 09月 21日		测试单位	试井队
矿 名	采油五矿		仪器名称	抽油井综合测试仪		分析结果	正常
冲 程	4.53 (m)	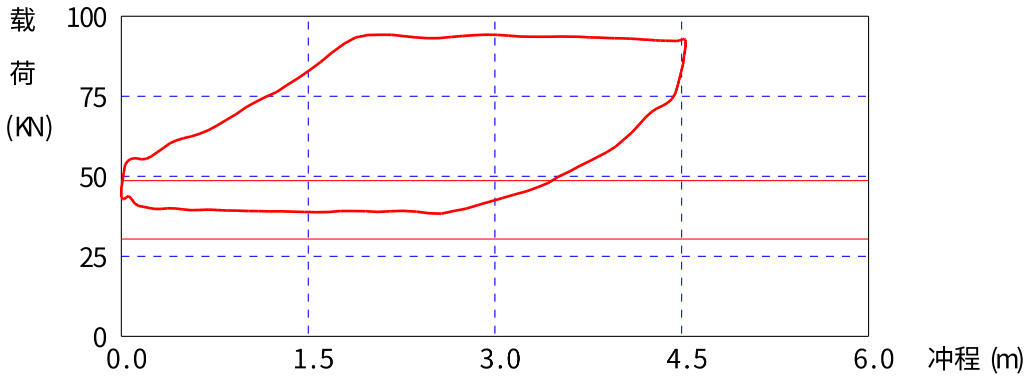					
冲 次	2.6 (min)						
上 载 荷	94.25 (KN)						
下 载 荷	38.33 (KN)						
泵 径	57 (mm)						
泵 深	927.19 (m)						
杆 径 一	28 (mm)						
杆 长 一	9.14 (m)						
杆 径 二	25 (mm)	液 柱 重	18.25 (KN)	实际产量	11 (t)	上 电 流	98 (A)
杆 长 二	907.53 (m)	杆 柱 重	30.4 (KN)	理论排量	42.25 (t)	下 电 流	72 (A)
杆 径 三	22 (mm)	油 压	0.46 (MPa)	含 水	83 (%)	动 液 面	132 (m)
杆 长 三	9.14 (m)	套 压	0.49 (MPa)	泵 效	26.04 (%)	沉 没 度	795.19 (m)
测 试 人	李 荣 华	计 算 人	盛 明 波	审 核 人	马 金 江	单位名称	第一采油厂

示 功 图 测 试 报 表

井　号	高162-50	测试日期	2016年 09月 22日	测试单位	试井队
矿　名	采油五矿	仪器名称	抽油井综合测试仪	分析结果	正常

冲　程	4.5	(m)
冲　次	2.6	(min)
上 载 荷	95.03	(KN)
下 载 荷	39.58	(KN)
泵　径	57	(mm)
泵　深	927.19	(m)
杆 径 一	28	(mm)
杆 长 一	9.14	(m)

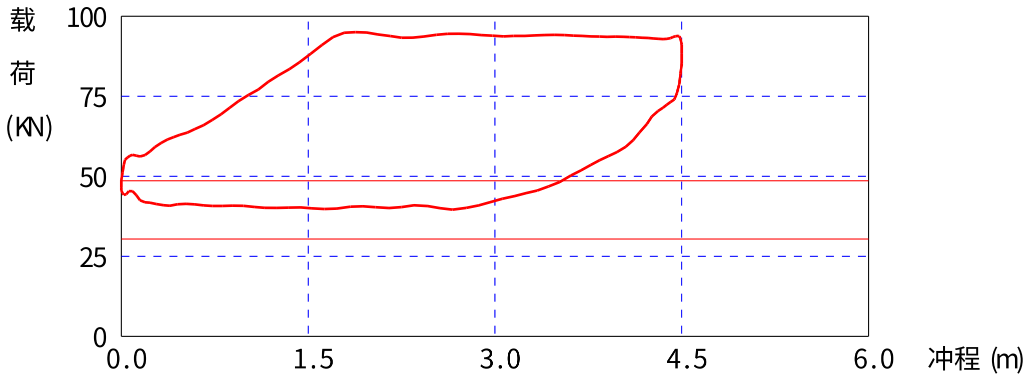

杆 径 二	25	(mm)	液 柱 重	18.2	(KN)	实际产量	11.24	(t)	上 电 流	97	(A)
杆 长 二	907.53	(m)	杆 柱 重	30.41	(KN)	理论排量	41.85	(t)	下 电 流	71	(A)
杆 径 三	22	(mm)	油　压	0.46	(MPa)	含　水	81	(%)	动 液 面	130.67	(m)
杆 长 三	9.14	(m)	套　压	0.49	(MPa)	泵　效	26.86	(%)	沉 没 度	796.52	(m)
测 试 人	李 荣 华		计 算 人	盛 明 波		审 核 人	马 金 江		单位名称	第一采油厂	

日 期 :2016-12-22 10:18 AM

示 功 图 测 试 报 表

井 号	高 162-50	测试日期	2016年 11月 04日	测试单位	试井队
矿 名	采油五矿	仪器名称	抽油井综合测试仪	分析结果	正常

冲 程	4.69	(m)
冲 次	2.5	(min)
上 载 荷	97.58	(KN)
下 载 荷	37.28	(KN)
泵 径	40	(mm)
泵 深	729.31	(m)
杆 径 一	28	(mm)
杆 长 一	9.14	(m)

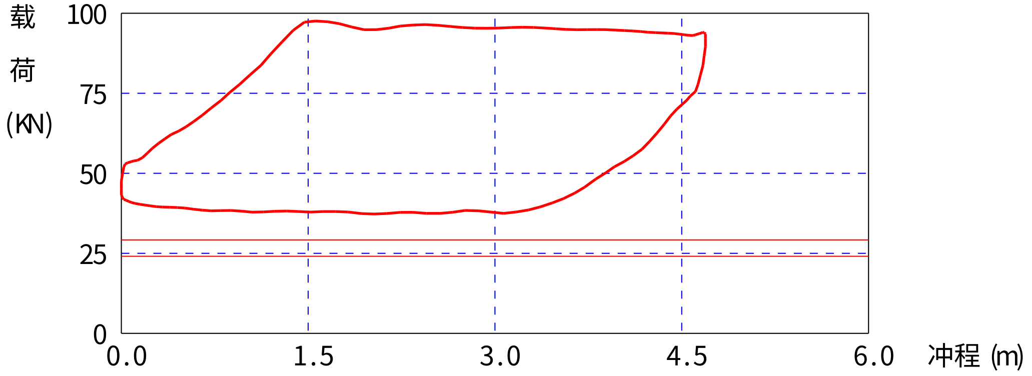

杆 径 二	25	(mm)	液 柱 重	5.11	(KN)	实际产量	4.53	(t)	上 电 流	117	(A)
杆 长 二	710.49	(m)	杆 柱 重	24.08	(KN)	理论排量	19.83	(t)	下 电 流	74	(A)
杆 径 三	22	(mm)	油 压	0.8	(MPa)	含 水	53.2	(%)	动 液 面	-1	(m)
杆 长 三	9.14	(m)	套 压	0.82	(MPa)	泵 效	22.85	(%)	沉 没 度	0	(m)
测 试 人	李 荣 华		计 算 人	盛 明 波		审 核 人	马 金 江		单位名称	第一采油厂	

日 期 :2016-12-22 10:18 AM

示　功　图　测　试　报　表

井　号	高 162-50		测试日期	2016年 10月 26日	测试单位	试井队	
矿　名	采油五矿		仪器名称	抽油井综合测试仪	分析结果	正常	
冲　程	4.67 (m)						
冲　次	2.5 (min)						
上载荷	99.38 (KN)						
下载荷	39.35 (KN)						
泵　径	40 (mm)						
泵　深	729.31 (m)						
杆径一	28 (mm)						
杆长一	9.14 (m)						
杆径二	25 (mm)	液柱重	5.05 (KN)	实际产量	5.65 (t)	上电流	116 (A)
杆长二	710.49 (m)	杆柱重	24.12 (KN)	理论排量	19.51 (t)	下电流	73 (A)
杆径三	22 (mm)	油　压	0.72 (MPa)	含　水	45.3 (%)	动液面	232 (m)
杆长三	9.14 (m)	套　压	0.78 (MPa)	泵　效	28.96 (%)	沉没度	497.31 (m)
测试人	李荣华	计算人	盛明波	审核人	马金江	单位名称	第一采油厂

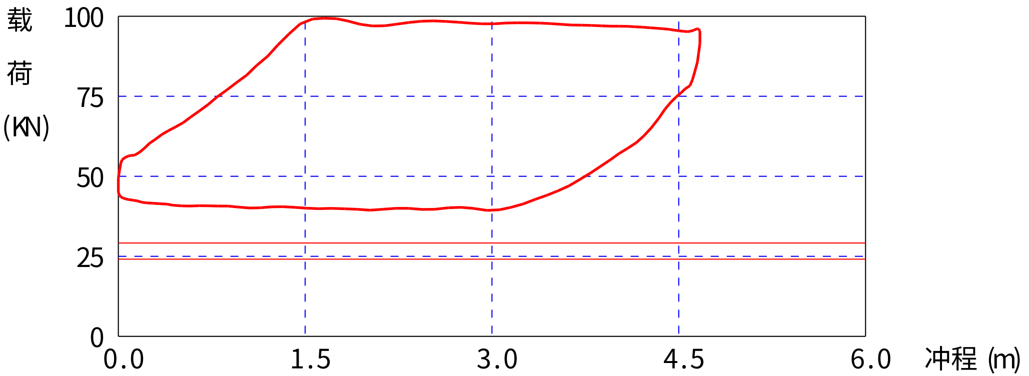

示 功 图 测 试 报 表

井 号	高 162-50	测试日期	2016年 11月 10日	测试单位	试井队
矿 名	采油五矿	仪器名称	抽油井综合测试仪	分析结果	正常

冲 程	5 (m)
冲 次	2.5 (min)
上 载 荷	97.65 (KN)
下 载 荷	39.97 (KN)
泵 径	40 (mm)
泵 深	729.31 (m)
杆 径 一	28 (mm)
杆 长 一	710.49 (m)

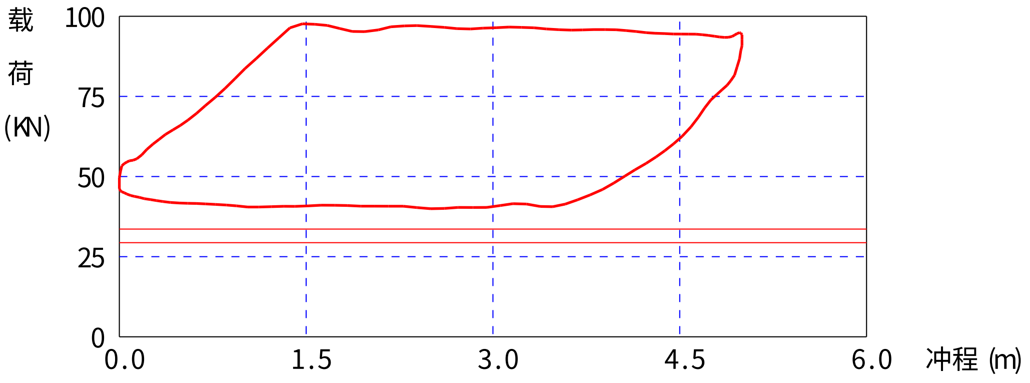

杆 径 二	0 (mm)	液 柱 重	4.24 (KN)	实际产量	6.2 (t)	上 电 流	118 (A)
杆 长 二	0 (m)	杆 柱 重	29.37 (KN)	理论排量	21.48 (t)	下 电 流	75 (A)
杆 径 三	0 (mm)	油 压	0.8 (MPa)	含 水	64 (%)	动 液 面	-1 (m)
杆 长 三	0 (m)	套 压	0.82 (MPa)	泵 效	28.86 (%)	沉 没 度	0 (m)
测 试 人	李 荣 华	计 算 人	盛 明 波	审 核 人	马 金 江	单位名称	第一采油厂

日 期 :2016-12-22 10:18 AM

示 功 图 测 试 报 表

井　　号	高 162-50	测试日期	2016年 11月 05日	测试单位	试井队
矿　　名	采油五矿	仪器名称	抽油井综合测试仪	分析结果	正常

冲　　程	4.65	(m)
冲　　次	2.6	(min)
上 载 荷	99.48	(KN)
下 载 荷	39.77	(KN)
泵　　径	40	(mm)
泵　　深	729.31	(m)
杆 径 一	28	(mm)
杆 长 一	9.14	(m)

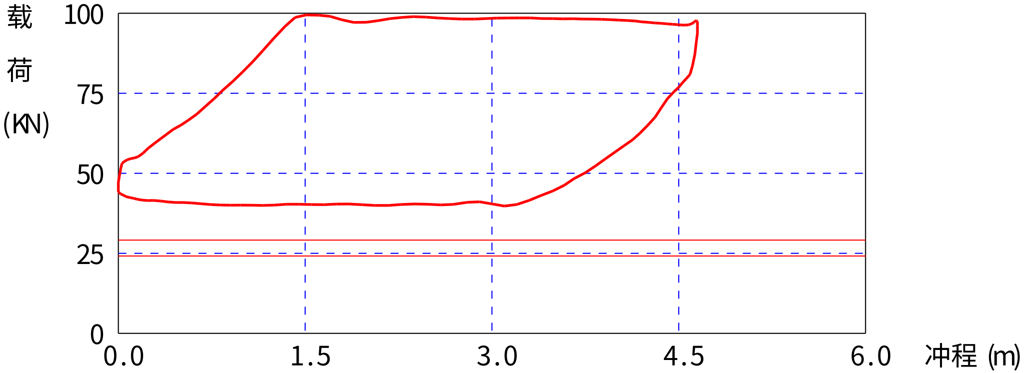

杆 径 二	25	(mm)	液 柱 重	4.96	(KN)	实际产量	4.43	(t)	上 电 流	114	(A)
杆 长 二	710.49	(m)	杆 柱 重	24.17	(KN)	理论排量	19.86	(t)	下 电 流	75	(A)
杆 径 三	22	(mm)	油　　压	0.8	(MPa)	含　　水	34	(%)	动 液 面	124.95	(m)
杆 长 三	9.14	(m)	套　　压	0.82	(MPa)	泵　　效	22.31	(%)	沉 没 度	604.36	(m)
测 试 人	李 荣 华		计 算 人	盛 明 波		审 核 人	马 金 江		单位名称	第一采油厂	

日 期 :2016-12-22 10:18 AM

示 功 图 测 试 报 表

井　　号	高162-50	测试日期	2016年11月20日	测试单位	试井队
矿　　名	采油五矿	仪器名称	抽油井综合测试仪	分析结果	正常

冲　　程	4.7 (m)
冲　　次	2.5 (min)
上 载 荷	98 (KN)
下 载 荷	36.85 (KN)
泵　　径	40 (mm)
泵　　深	729.31 (m)
杆 径 一	28 (mm)
杆 长 一	710.49 (m)

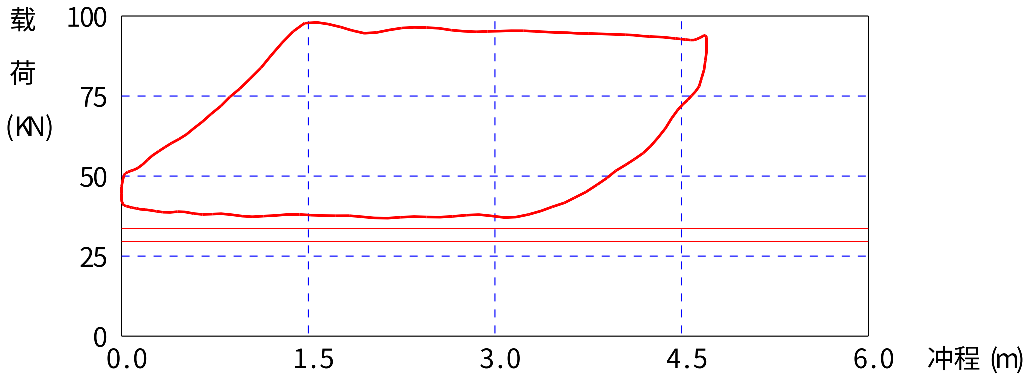

杆 径 二	0 (mm)	液 柱 重	4.1 (KN)	实际产量	6.3 (t)	上 电 流	119 (A)
杆 长 二	0 (m)	杆 柱 重	29.5 (KN)	理论排量	19.54 (t)	下 电 流	75 (A)
杆 径 三	0 (mm)	油　　压	0.82 (MPa)	含　　水	42 (%)	动 液 面	205.33 (m)
杆 长 三	0 (m)	套　　压	0.91 (MPa)	泵　　效	32.25 (%)	沉 没 度	523.98 (m)
测 试 人	李 荣 华	计 算 人	盛 明 波	审 核 人	马 金 江	单位名称	第一采油厂

日 期 :2016-12-22 10:18 AM

示 功 图 测 试 报 表

井 号	高 162-50	测试日期	2016年 11月 25日	测试单位	试井队
矿 名	采油五矿	仪器名称	抽油井综合测试仪	分析结果	正常

冲 程	4.75	(m)
冲 次	2.5	(min)
上 载 荷	95.06	(KN)
下 载 荷	37.28	(KN)
泵 径	40	(mm)
泵 深	729.31	(m)
杆 径 一	28	(mm)
杆 长 一	710.49	(m)

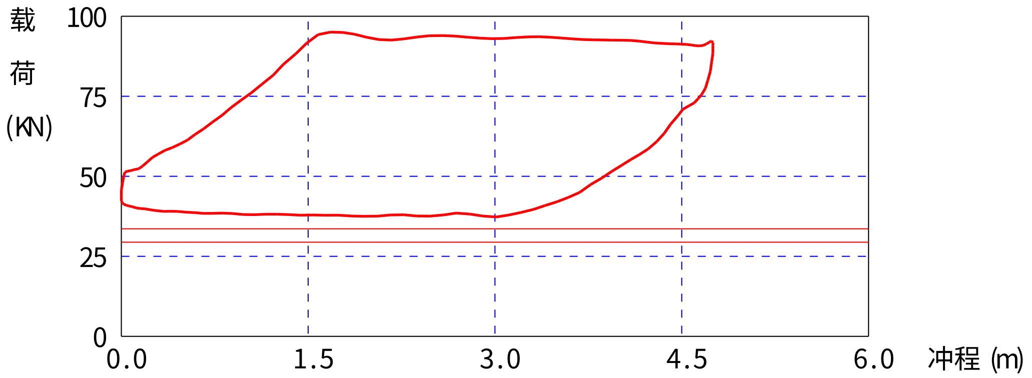

杆 径 二	0	(mm)	液 柱 重	4.18	(KN)	实际产量	6.9	(t)	上 电 流	117	(A)
杆 长 二	0	(m)	杆 柱 重	29.42	(KN)	理论排量	20.14	(t)	下 电 流	74	(A)
杆 径 三	0	(mm)	油 压	0.62	(MPa)	含 水	55.1	(%)	动 液 面	170.67	(m)
杆 长 三	0	(m)	套 压	0.71	(MPa)	泵 效	34.26	(%)	沉 没 度	558.64	(m)
测 试 人	李 荣 华		计 算 人	盛 明 波		审 核 人	马 金 江		单位名称	第一采油厂	

日 期 :2016-12-22 10:18 AM

示 功 图 测 试 报 表

井号	高 162-50	测试日期	2016年 12月 06日	测试单位	试井队
矿名	采油五矿	仪器名称	抽油井综合测试仪	分析结果	正常

冲程	4.72	(m)
冲次	2.9	(min)
上载荷	98.39	(KN)
下载荷	38.22	(KN)
泵径	40	(mm)
泵深	729.31	(m)
杆径一	28	(mm)
杆长一	710.49	(m)

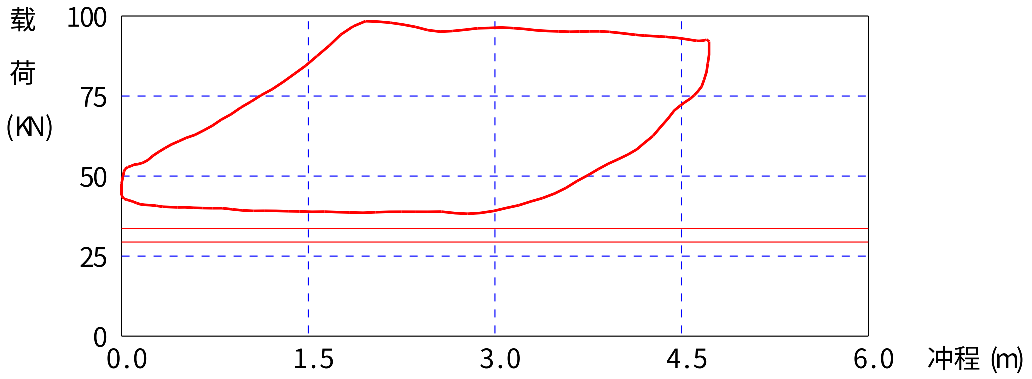

杆径二	0 (mm)	液柱重	4.22 (KN)	实际产量	9.81 (t)	上电流	115 (A)
杆长二	0 (m)	杆柱重	29.39 (KN)	理论排量	23.42 (t)	下电流	72 (A)
杆径三	0 (mm)	油压	0.42 (MPa)	含水	61 (%)	动液面	159.47 (m)
杆长三	0 (m)	套压	0.51 (MPa)	泵效	41.89 (%)	沉没度	569.84 (m)
测试人	李荣华	计算人	盛明波	审核人	马金江	单位名称	第一采油厂

示 功 图 测 试 报 表

井　号	高 162-50	测试日期	2016年 11月 29日	测试单位	试井队
矿　名	采油五矿	仪器名称	抽油井综合测试仪	分析结果	正常

冲　程	4.75	(m)
冲　次	2.5	(min)
上载荷	95.61	(KN)
下载荷	37.46	(KN)
泵　径	40	(mm)
泵　深	729.31	(m)
杆径一	28	(mm)
杆长一	710.49	(m)

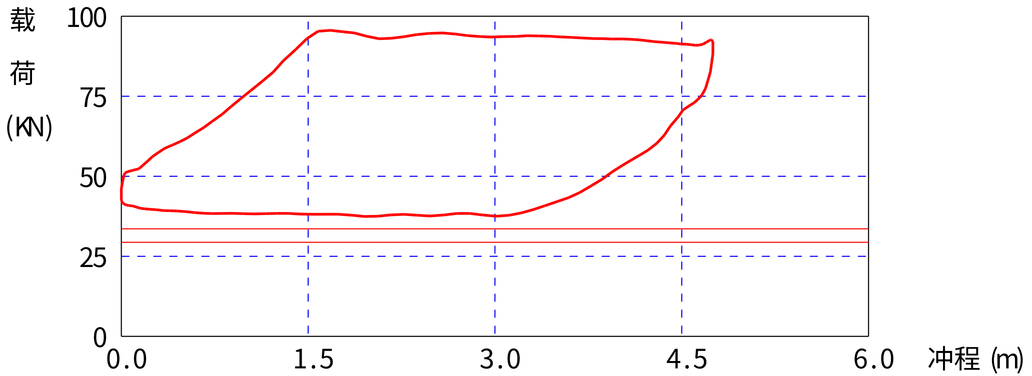

杆径二	0 (mm)	液柱重	4.22 (KN)	实际产量	7.3 (t)	上电流	116 (A)
杆长二	0 (m)	杆柱重	29.38 (KN)	理论排量	20.34 (t)	下电流	72 (A)
杆径三	0 (mm)	油　压	0.62 (MPa)	含　水	61.8 (%)	动液面	205.33 (m)
杆长三	0 (m)	套　压	0.71 (MPa)	泵　效	35.89 (%)	沉没度	523.98 (m)
测试人	李荣华	计算人	盛明波	审核人	马金江	单位名称	第一采油厂

日 期 :2016-12-22 10:18 AM

示 功 图 测 试 报 表

井　　号	高 162-50	测试日期	2016年 11月 16日	测试单位	试井队
矿　　名	采油五矿	仪器名称	抽油井综合测试仪	分析结果	正常

冲　　程	4.68 (m)
冲　　次	2.5 (min)
上 载 荷	96.52 (KN)
下 载 荷	36.85 (KN)
泵　　径	40 (mm)
泵　　深	729.31 (m)
杆 径 一	28 (mm)
杆 长 一	710.49 (m)

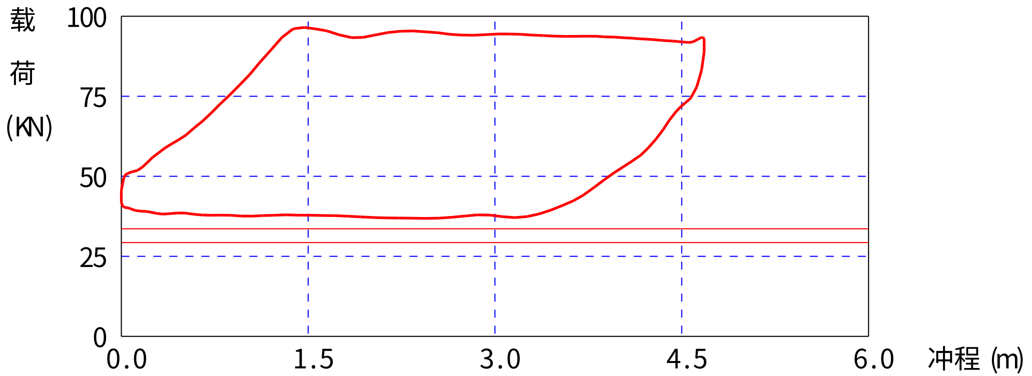

杆 径 二	0 (mm)	液 柱 重	4.31 (KN)	实际产量	5.5 (t)	上 电 流	117 (A)
杆 长 二	0 (m)	杆 柱 重	29.3 (KN)	理论排量	20.46 (t)	下 电 流	73 (A)
杆 径 三	0 (mm)	油　　压	0.82 (MPa)	含　　水	76.1 (%)	动 液 面	197.33 (m)
杆 长 三	0 (m)	套　　压	0.91 (MPa)	泵　　效	26.88 (%)	沉 没 度	531.98 (m)
测 试 人	李 荣 华	计 算 人	盛 明 波	审 核 人	马 金 江	单位名称	第一采油厂

日 期 :2016-12-22 10:18 AM

示 功 图 测 试 报 表

井号	高 162-50	测试日期	2016年 11月 13日	测试单位	试井队
矿名	采油五矿	仪器名称	抽油井综合测试仪	分析结果	正常

冲程	4.86	(m)
冲次	2.5	(min)
上载荷	98.22	(KN)
下载荷	37.24	(KN)
泵径	40	(mm)
泵深	729.31	(m)
杆径一	28	(mm)
杆长一	710.49	(m)

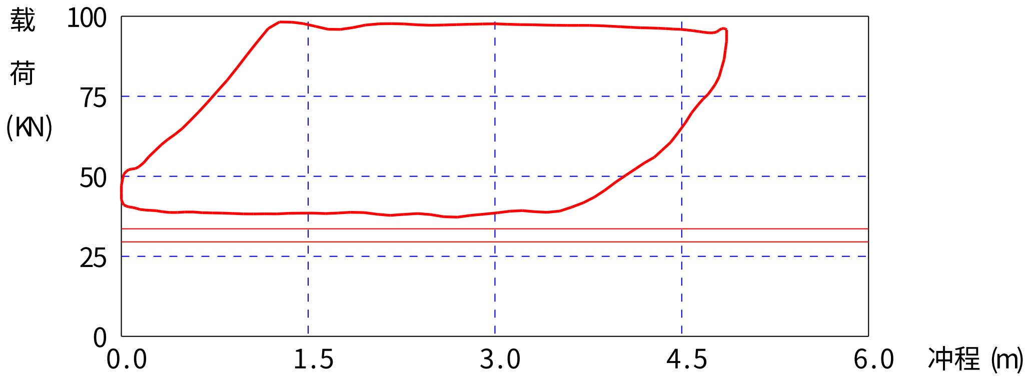

杆径二	0	(mm)	液柱重	4.08	(KN)	实际产量	3.58	(t)	上电流	118	(A)
杆长二	0	(m)	杆柱重	29.52	(KN)	理论排量	20.11	(t)	下电流	73	(A)
杆径三	0	(mm)	油压	0.82	(MPa)	含水	39	(%)	动液面	202.67	(m)
杆长三	0	(m)	套压	0.91	(MPa)	泵效	17.8	(%)	沉没度	526.64	(m)
测试人	李荣华		计算人	盛明波		审核人	马金江		单位名称	第一采油厂	

日 期 :2016-12-22 10:18 AM

示 功 图 测 试 报 表

井　　号	高 162-50	测试日期	2016年 11月 27日	测试单位	试井队
矿　　名	采油五矿	仪器名称	抽油井综合测试仪	分析结果	正常

冲　　程	4.73	(m)
冲　　次	2.5	(min)
上 载 荷	95.14	(KN)
下 载 荷	36.76	(KN)
泵　　径	40	(mm)
泵　　深	729.31	(m)
杆 径 一	28	(mm)
杆 长 一	710.49	(m)

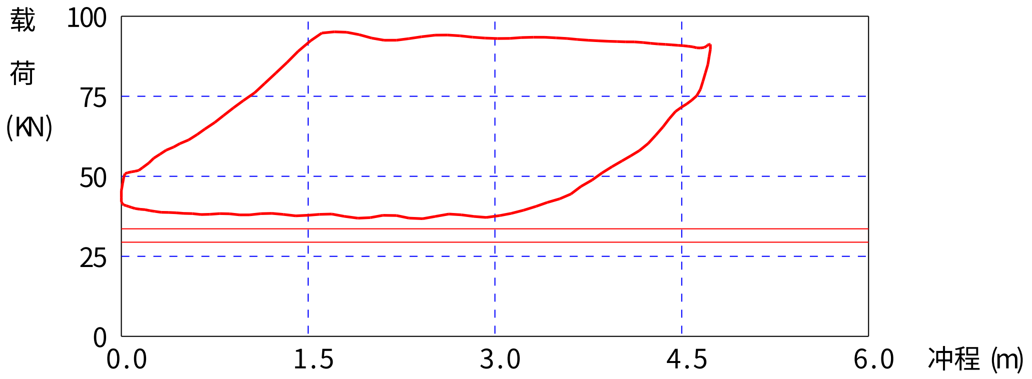

杆 径 二	0	(mm)	液 柱 重	4.18	(KN)	实际产量	7	(t)	上 电 流	119	(A)
杆 长 二	0	(m)	杆 柱 重	29.42	(KN)	理论排量	20.05	(t)	下 电 流	76	(A)
杆 径 三	0	(mm)	油　　压	0.62	(MPa)	含　　水	55	(%)	动 液 面	198.67	(m)
杆 长 三	0	(m)	套　　压	0.71	(MPa)	泵　　效	34.91	(%)	沉 没 度	530.64	(m)
测 试 人	李 荣 华		计 算 人	盛 明 波		审 核 人	马 金 江		单位名称	第一采油厂	

日 期 :2016-12-22 10:18 AM

示 功 图 测 试 报 表

井 号	高 162-50	测试日期	2016年 12月 07日	测试单位	试井队
矿 名	采油五矿	仪器名称	抽油井综合测试仪	分析结果	正常

冲 程	4.74	(m)
冲 次	2.8	(min)
上 载 荷	97.06	(KN)
下 载 荷	38.97	(KN)
泵 径	40	(mm)
泵 深	729.31	(m)
杆 径 一	28	(mm)
杆 长 一	710.49	(m)

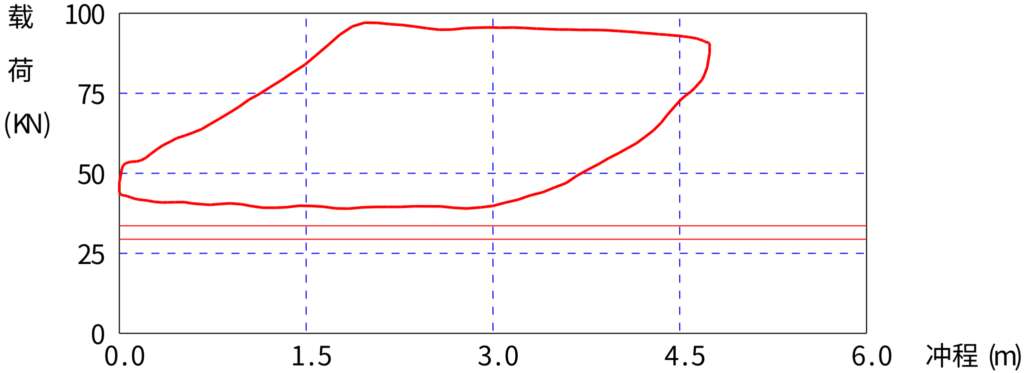

杆 径 二	0	(mm)	液 柱 重	4.21	(KN)	实际产量	11.2	(t)	上 电 流	119	(A)
杆 长 二	0	(m)	杆 柱 重	29.4	(KN)	理论排量	22.65	(t)	下 电 流	74	(A)
杆 径 三	0	(mm)	油 压	0.42	(MPa)	含 水	59.3	(%)	动 液 面	179.96	(m)
杆 长 三	0	(m)	套 压	0.51	(MPa)	泵 效	49.45	(%)	沉 没 度	549.35	(m)
测 试 人	李 荣 华		计 算 人	盛 明 波		审 核 人	马 金 江		单位名称	第一采油厂	

日 期 :2016-12-22 10:18 AM

示 功 图 测 试 报 表

井　　号	高 162-50	测试日期	2016年 12月 15日	测试单位	试井队
矿　　名	采油五矿	仪器名称	抽油井综合测试仪	分析结果	正常

冲　　程	4.83	(m)
冲　　次	2.8	(min)
上 载 荷	98.67	(KN)
下 载 荷	37.51	(KN)
泵　　径	40	(mm)
泵　　深	729.31	(m)
杆 径 一	28	(mm)
杆 长 一	710.49	(m)

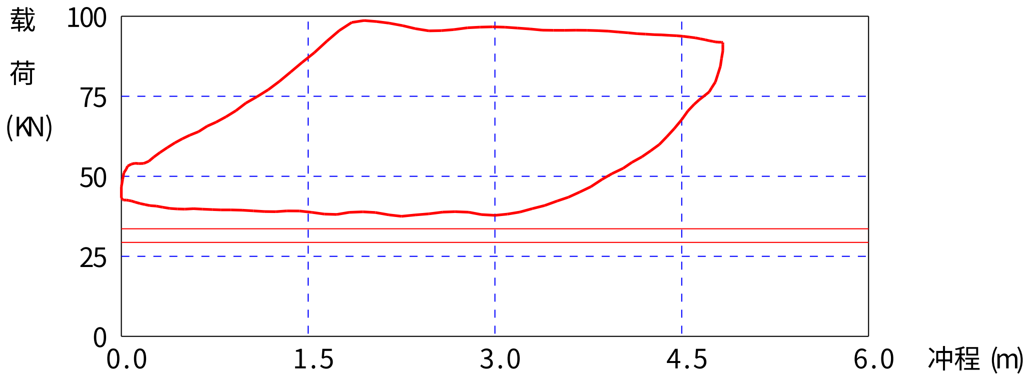

杆 径 二	0	(mm)	液 柱 重	4.26	(KN)	实际产量	11.07	(t)	上 电 流	126	(A)
杆 长 二	0	(m)	杆 柱 重	29.35	(KN)	理论排量	23.37	(t)	下 电 流	81	(A)
杆 径 三	0	(mm)	油　　压	0.45	(MPa)	含　　水	67.7	(%)	动 液 面	-1	(m)
杆 长 三	0	(m)	套　　压	0.46	(MPa)	泵　　效	47.38	(%)	沉 没 度	0	(m)
测 试 人	李 荣 华		计 算 人	盛 明 波		审 核 人	马 金 江		单位名称	第一采油厂	

示 功 图 测 试 报 表

井 号	高 162-50	测试日期	2016年 12月 09日	测试单位	试井队
矿 名	采油五矿	仪器名称	抽油井综合测试仪	分析结果	正常

冲 程	4.79	(m)
冲 次	2.9	(min)
上 载 荷	97.38	(KN)
下 载 荷	38.65	(KN)
泵 径	40	(mm)
泵 深	729.31	(m)
杆 径 一	28	(mm)
杆 长 一	710.49	(m)

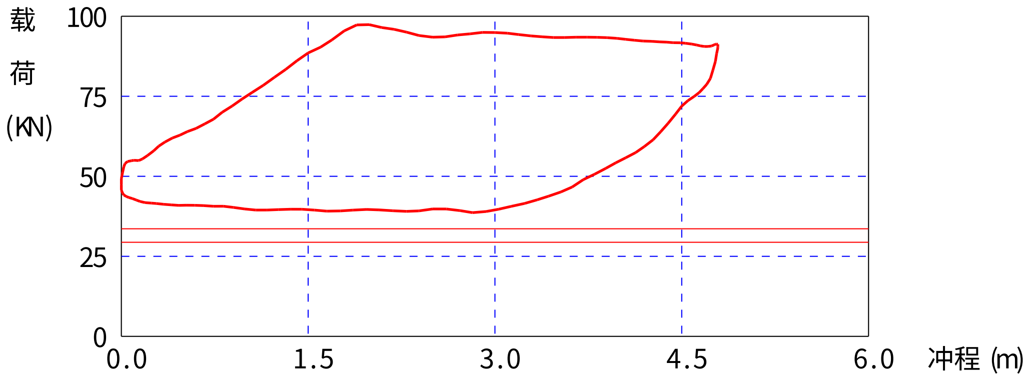

杆 径 二	0 (mm)	液 柱 重	4.22 (KN)	实际产量	11.8 (t)	上 电 流	117 (A)
杆 长 二	0 (m)	杆 柱 重	29.39 (KN)	理论排量	23.77 (t)	下 电 流	73 (A)
杆 径 三	0 (mm)	油 压	0.42 (MPa)	含 水	61.2 (%)	动 液 面	172 (m)
杆 长 三	0 (m)	套 压	0.51 (MPa)	泵 效	49.64 (%)	沉 没 度	557.31 (m)
测 试 人	李 荣 华	计 算 人	盛 明 波	审 核 人	马 金 江	单位名称	第一采油厂

示 功 图 测 试 报 表

井　　号	高 162-50	测试日期	2016年 12月 16日	测试单位	试井队
矿　　名	采油五矿	仪器名称	抽油井综合测试仪	分析结果	正常

冲　　程	4.83	(m)
冲　　次	2.9	(min)
上 载 荷	97.74	(KN)
下 载 荷	37.54	(KN)
泵　　径	40	(mm)
泵　　深	729.31	(m)
杆 径 一	28	(mm)
杆 长 一	710.49	(m)

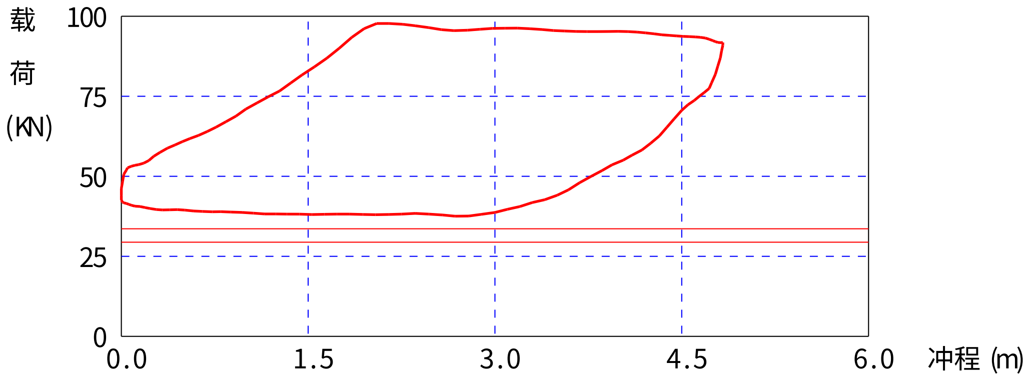

杆 径 二	0	(mm)	液 柱 重	4.19	(KN)	实际产量	11.07	(t)	上 电 流	121	(A)
杆 长 二	0	(m)	杆 柱 重	29.42	(KN)	理论排量	23.78	(t)	下 电 流	76	(A)
杆 径 三	0	(mm)	油　　压	0.45	(MPa)	含　　水	55.8	(%)	动 液 面	186.67	(m)
杆 长 三	0	(m)	套　　压	0.46	(MPa)	泵　　效	46.56	(%)	沉 没 度	542.64	(m)
测 试 人	李 荣 华		计 算 人	盛 明 波		审 核 人	马 金 江		单位名称	第一采油厂	

示 功 图 测 试 报 表

井 号	高 162-50		测试日期	2016年 12月 05日	测试单位	试井队	
矿 名	采油五矿		仪器名称	抽油井综合测试仪	分析结果	正常	
冲 程	4.7 (m)						
冲 次	2.9 (min)						
上 载 荷	98.01 (KN)						
下 载 荷	37.35 (KN)						
泵 径	40 (mm)						
泵 深	729.31 (m)						
杆 径 一	28 (mm)						
杆 长 一	710.49 (m)						
杆 径 二	0 (mm)	液 柱 重	4.22 (KN)	实际产量	9.9 (t)	上 电 流	115 (A)
杆 长 二	0 (m)	杆 柱 重	29.38 (KN)	理论排量	23.34 (t)	下 电 流	74 (A)
杆 径 三	0 (mm)	油 压	0.42 (MPa)	含 水	61.6 (%)	动 液 面	189.15 (m)
杆 长 三	0 (m)	套 压	0.51 (MPa)	泵 效	42.42 (%)	沉 没 度	540.16 (m)
测 试 人	李 荣 华	计 算 人	盛 明 波	审 核 人	马 金 江	单位名称	第一采油厂

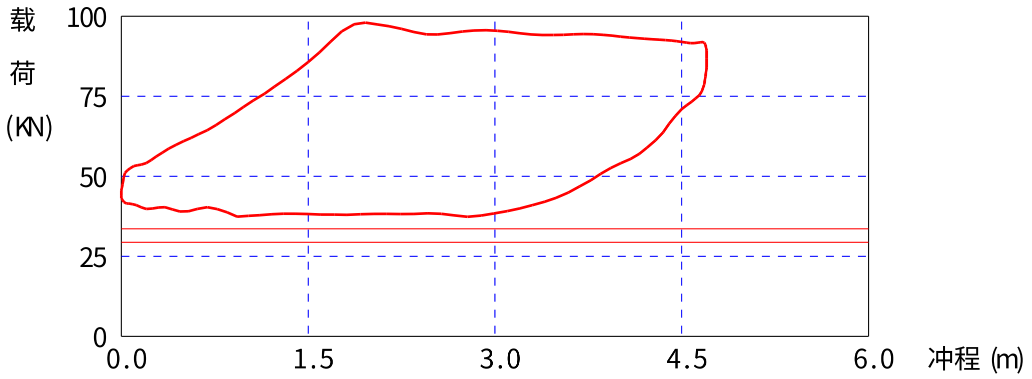

日 期 :2016-12-22 10:18 AM

示 功 图 测 试 报 表

井 号	高 162-50	测试日期	2016年 12月 20日	测试单位	试井队
矿 名	采油五矿	仪器名称	抽油井综合测试仪	分析结果	正常

冲 程	4.82	(m)
冲 次	2.9	(min)
上 载 荷	96.46	(KN)
下 载 荷	39.15	(KN)
泵 径	40	(mm)
泵 深	729.31	(m)
杆 径 一	28	(mm)
杆 长 一	710.49	(m)

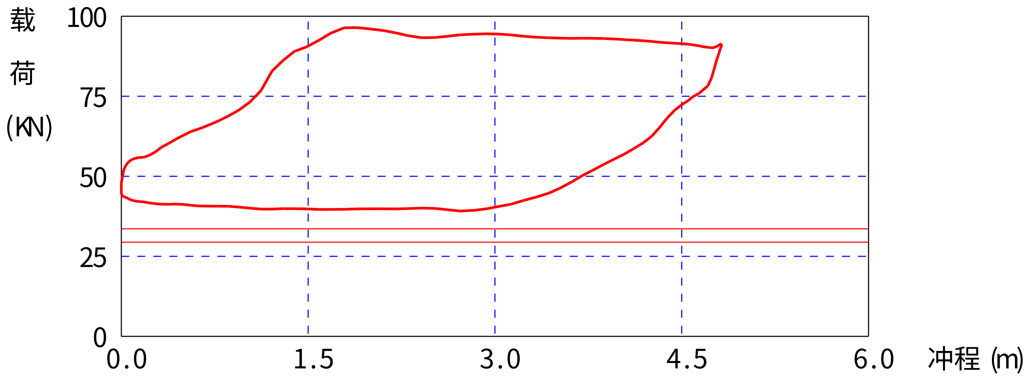

杆 径 二	0	(mm)	液 柱 重	4.18	(KN)	实际产量	10.81	(t)	上 电 流	117	(A)
杆 长 二	0	(m)	杆 柱 重	29.42	(KN)	理论排量	23.7	(t)	下 电 流	76	(A)
杆 径 三	0	(mm)	油 压	0.45	(MPa)	含 水	54.9	(%)	动 液 面	170.67	(m)
杆 长 三	0	(m)	套 压	0.46	(MPa)	泵 效	45.62	(%)	沉 没 度	558.64	(m)
测 试 人	李 荣 华		计 算 人	盛 明 波		审 核 人	马 金 江		单位名称	第一采油厂	

日 期 :2016-12-22 10:18 AM

示 功 图 测 试 报 表

井　　号	高 162-50	测试日期	2016年 12月 21日	测试单位	试井队
矿　　名	采油五矿	仪器名称	抽油井综合测试仪	分析结果	正常

冲　　程	4.85	(m)
冲　　次	2.8	(min)
上 载 荷	97.58	(KN)
下 载 荷	40.35	(KN)
泵　　径	40	(mm)
泵　　深	729.31	(m)
杆 径 一	28	(mm)
杆 长 一	710.49	(m)

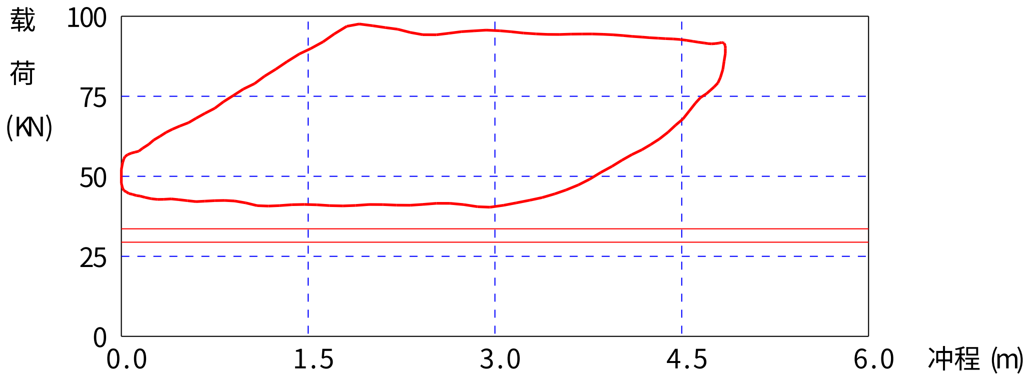

杆 径 二	0 (mm)	液 柱 重	4.18 (KN)	实际产量	10.48 (t)	上 电 流	119 (A)
杆 长 二	0 (m)	杆 柱 重	29.42 (KN)	理论排量	23.02 (t)	下 电 流	75 (A)
杆 径 三	0 (mm)	油　　压	0.44 (MPa)	含　　水	54.9 (%)	动 液 面	118.67 (m)
杆 长 三	0 (m)	套　　压	0.48 (MPa)	泵　　效	45.52 (%)	沉 没 度	610.64 (m)
测 试 人	李 荣 华	计 算 人	盛 明 波	审 核 人	马 金 江	单位名称	第一采油厂

日 期 :2016-12-22 10:18 AM
